# Mother Centriole Distal Appendages Mediate Centrosome Docking at the Immunological Synapse and Reveal Mechanistic Parallels with Ciliogenesis

**DOI:** 10.1016/j.cub.2015.10.028

**Published:** 2015-12-21

**Authors:** Jane C. Stinchcombe, Lyra O. Randzavola, Karen L. Angus, Judith M. Mantell, Paul Verkade, Gillian M. Griffiths

**Affiliations:** 1 Cambridge Institute for Medical Research, Cambridge Biomedical Campus, Hills Road, Cambridge CB2 0XY, UK; 2 Wolfson Bioimaging Facility, University of Bristol, Medical Sciences, Bristol BS8 1TD, UK

## Abstract

Cytotoxic T lymphocytes (CTLs) are highly effective serial killers capable of destroying virally infected and cancerous targets by polarized release from secretory lysosomes. Upon target contact, the CTL centrosome rapidly moves to the immunological synapse, focusing microtubule-directed release at this point [1, 2, 3]. Striking similarities have been noted between centrosome polarization at the synapse and basal body docking during ciliogenesis [1, 4, 5, 6, 7, 8], suggesting that CTL centrosomes might dock with the plasma membrane during killing, in a manner analogous to primary cilia formation [1, 4]. However, questions remain regarding the extent and function of centrosome polarization at the synapse, and recent reports have challenged its role [9, 10]. Here, we use high-resolution transmission electron microscopy (TEM) tomography analysis to show that, as in ciliogenesis, the distal appendages of the CTL mother centriole contact the plasma membrane directly during synapse formation. This is functionally important as small interfering RNA (siRNA) targeting of the distal appendage protein, Cep83, required for membrane contact during ciliogenesis [11], impairs CTL secretion. Furthermore, the regulatory proteins CP110 and Cep97, which must dissociate from the mother centriole to allow cilia formation [12], remain associated with the mother centriole in CTLs, and neither axoneme nor transition zone ciliary structures form. Moreover, complete centrosome docking can occur in proliferating CTLs with multiple centriole pairs. Thus, in CTLs, centrosomes dock transiently with the membrane, within the cell cycle and without progression into ciliogenesis. We propose that this transient centrosome docking without cilia formation is important for CTLs to deliver rapid, repeated polarized secretion directed by the centrosome.

## Highlights

• Centrosome docking at the immune synapse parallels early ciliogenesis
• CTL mother centriole distal appendages dock directly with the membrane
• Cep83 depletion inhibits CTL secretion
• CP110 is retained on the mother centrioles and ciliogenesis is blocked

## Graphical Abstract

**Figure:**
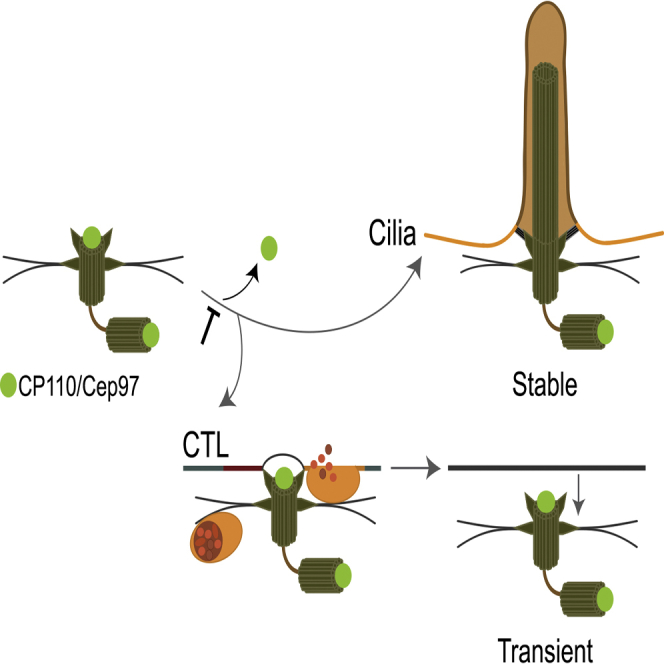

## Teaser

Stinchcombe et al. show that centrosome docking at the immunological synapse of CTLs mirrors early ciliogenesis, with the mother centriole docking via distal appendages, but further ciliogenesis blocked. The authors propose that transient centrosome docking without ciliogenesis will favor the rapid serial killing used by CTLs.

## Results and Discussion

Activated CTLs proliferate rapidly, suggesting that some CTLs may contain replicating centrioles. Although ciliogenesis is often reported to occur during the G0-G1 transition and require exit from the cell cycle [13, 14, 15], there are several instances of cilia formation in cycling and differentiated cells [16]. This suggested centrosome polarization in CTLs might continue throughout activated populations, regardless of cell-cycle state. To address this, we investigated whether centrosome docking can occur in proliferating CTLs undergoing centriole duplication.

**Figure 1:**
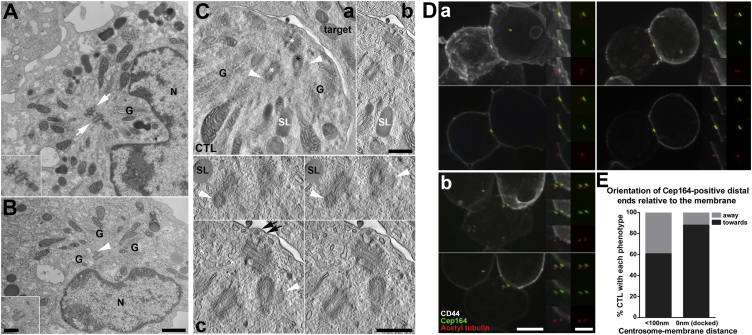
CTLs Contain Multiple Centriole Pairs, which All Polarize toward the Target with a Mother Centriole nearest the Membrane (A and B) 100–200 nm (A) or 50–100 nm (B) TEM sections from C57BL/6 CTLs with more than one appendage-bearing mother centriole (white arrows and inset, A) or centrioles with associated procentrioles (white arrowhead and inset, B). G, Golgi; N, nucleus. Scale bars, 1 μm and 500 nm (insets). See also Figure S1. (C) Multiple (Ca) or paired (Cb and Cc) plane projection images from a TEM tomograph of an F5 CTL-EL4 target synapse where the CTL contains more than one distal-appendage-bearing mother centriole (white asterisks, Ca) and centrioles bearing procentrioles (white arrowheads, Ca and Cc). Black arrows, fibrous connections between sub-distal appendages and the membrane; SL, secretory lysosome; white SL, secretory lysosome associated with microtubules; G, Golgi. Scale bars, 500 nm. See also Movie S1. (D) Confocal projection (top) or single-plane (bottom) images of OT-I CTLs (left) conjugated to EL4 targets (right), labeled with acetylated tubulin (centrioles, red), Cep164 (mother centriole distal appendages, green), and CD44 (plasma membrane of both CTLs and EL4 targets, white). CTLs with one (Da) or two (Db) tightly polarized mother centrioles are shown. Scale bars, 5 μm and 2.5 μm (insets). (E) Quantitation of mother centriole orientation at the CTL membrane in conjugates, assessed by the proximity and direction of Cep164-positive distal ends relative to the membrane.

EM analysis of CTLs (Figures 1A and 1B) revealed the presence of cells with procentriole-bearing centrioles, consistent with replication events (Figure 1B). Curiously, we also observed cells with more than one complete mature centriole pair, indicated by cells with multiple pairs, each with an appendage-bearing mother centriole (Figure 1A). To determine the frequency of CTLs with multiple or replicating centriole pairs, we fixed CTLs and labeled them for immunofluorescence with antibodies against acetylated tubulin, gamma tubulin, or centrin-3 to identify both mother and daughter centrioles and either CP110 or Cep97 to identify the distal ends of all centrioles and procentrioles [12, 17] or Cep164, a mother centriole distal appendage protein [18] (Figures S1 and S2). Using this approach, more than two CP110- or Cep97-positive structures per cell identified multiple centriole pairs and replicating centrioles while more than one Cep164-positive structure identified more than one mature mother centriole. CTLs with two or more Cep164-positive or with two, four, six, eight, or, occasionally, more than eight CP110- or Cep97-positive structures were all observed (Figures S1A–S1C). Quantitation of CP110- (n = 317) or Cep164- (n = 347) labeled CTLs revealed 32% contained more than two CP110-labeled structures, while 14% contained more than one Cep164-labeled ring (Figure S1D). Thus, one-third of CTLs within the population showed centriole duplication and/or more than one pair of centrioles.

As so many CTLs possessed multiple or replicating centrioles, we asked whether cells with replicating, or more than one pair of, centrioles could polarize their centrosome to the synapse during interaction with a target. TEM tomography revealed that CTLs polarize their centrioles to the plasma membrane regardless of centriole number or state of replication (Figure 1C; Movie S1). In CTLs with more than one centriole pair, all centrioles were polarized at the synapse, but only one pair was closely associated with the plasma membrane, with the additional pairs lying just behind the leading pair (Figure 1C; Movie S1).

To determine the frequency at which duplicating centrioles dock, we labeled CTL-target conjugates with antibodies against centrin-3 to identify both mother and daughter centrioles and either CP110 or mother centriole-specific Cep164. 10% Cep164-labeled CTLs (n = 320) contained more than one Cep164-labeled centriole, while 45% CP110-labeled cells (n = 435) had more than two CP110-labeled centrioles. CP110 association did not change upon CTL interaction with targets, with more than two CP110-labeled centrioles in 43% CTLs without targets (n = 110), 44% target-interacting CTLs (centrosome >0.5 μm from synapse, n = 137), and 47% polarized CTLs (centrosome <0.5 μm from synapse; n = 188) (Figure S1E). CTLs with more than one Cep164-labeled centriole increased upon centrosome polarization from 3% in CTLs without targets (n = 76), 7% unpolarized target-conjugated CTLs (n = 89) to 16% polarized conjugated CTLs (n = 155). Although the significance of this increase in polarized cells is not clear, our results show neither replication state nor multiple centrioles prevented polarization of CTL centrioles. Thus, CTLs do not need to exit the cell cycle for centrosome polarization.

Cilia and flagellar basal bodies dock directly at the plasma membrane, with the mother centriole attached via the distal appendages and the daughter centriole behind [19, 20]. We noted that Cep164 and CD44 plasma membrane marker labeling often overlapped (Figure 1D), suggesting that CTL centrosomes might adopt a specific topology at the membrane. Quantitation revealed the Cep164-labeled ends of the mother centriole faced the plasma membrane in 84% of CTL-target conjugates with docked centrosomes (n = 51) (Figure 1E). This suggested that the distal end of the mother centriole might be preferentially orientated toward the membrane when contacting the plasma membrane.

**Figure 2:**
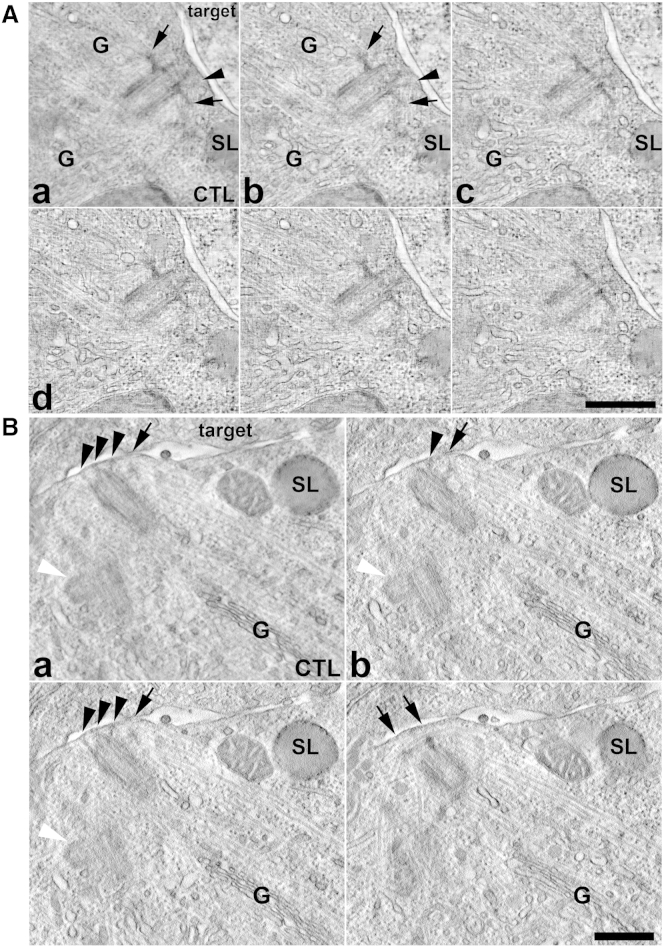
Centrosome Docking at the CTL Immunological Synapse Resembles Basal Body Docking during Ciliogenesis Multiple (Aa–Ac and Ba) or paired (Ad, Bc, and Bd) projection images from dual- (A) or single- (B) axis TEM tomographic reconstructions of synapses formed between F5 CTL (CTL) and EL4 (target) cells revealing (A) the orientation of the polarized mother centriole distal appendages (black arrowheads) and sub-distal appendages (black arrows) at the plasma membrane; (B) CTL mother centrioles dock with the synapse membrane via their distal appendages (black arrowheads), with microtubules radiating out from the sub-distal appendages (black arrows). White arrowheads, procentriole associated with polarized centriole. SL, secretory lysosome; G, Golgi. Scale bars, 500 nm. See also Movies S2 and S3.

**Figure 3:**
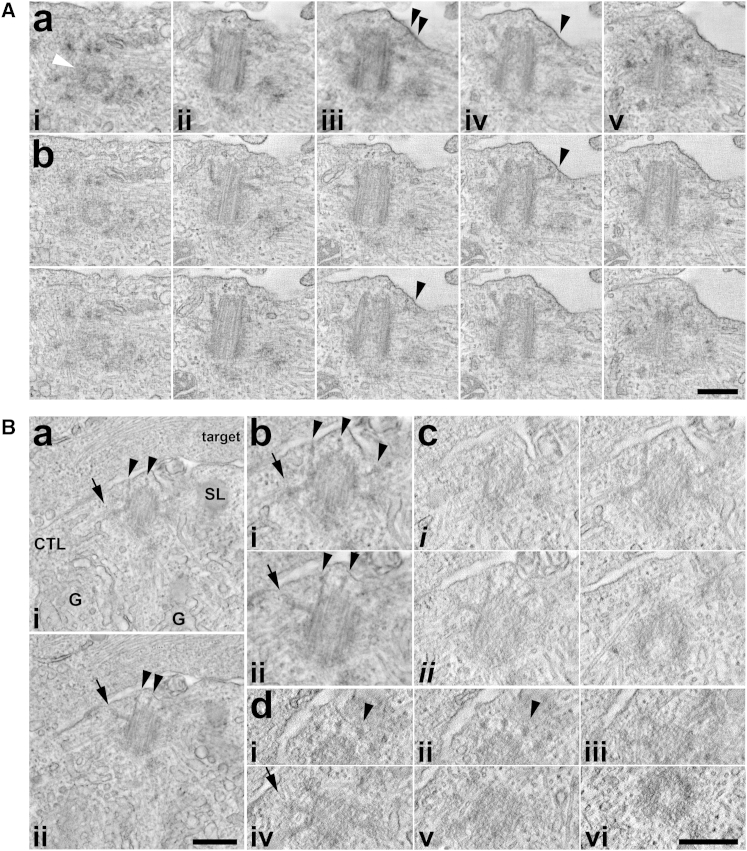
Docked Mother Centrioles Do Not Form a Transition Zone or Axoneme Images from (A) a dual-axis tilt series tomogram of a C57BL/6 CTL-P815 synapse and (B) a single-axis tilt series tomogram of an F5 CTL-EL4 target synapse, shown as multiple (Aa, Ba, and Bb) or paired (Ab, Bc, and Bd) plane projection images aligned (A and Ba–Bc) or tilted (Bd) to the axis of reconstruction. Arrowheads, distal appendages; arrows, sub-distal appendages; G, Golgi complex; SL, secretory lysosome. Scale bars, 300 nm. See also Movies S4 (A), S5 (Ba), and S6 (Bb and Bc).

TEM tomography was used to dissect the polarized centrosome topology in 3D at high resolution. Tomograms of docked centrosomes (n = 9) revealed the mother centriole contacted the plasma membrane directly via the ends of the distal appendages (Figure 2, arrowheads; Movies S2 and S3). The orientation of the docked mother centriole organized microtubules emanating from the sub-distal appendages (arrows) to lie directly underneath the synapse membrane (Figure 2A; Movie S2). 3D reconstructions revealed that most of these microtubules were long, extending directly from the docking site back into the cell body (Figures 2A and 2B; Movies S2 and S3). Secretory lysosomes and Golgi cisternae were aligned along microtubules emanating from the polarized centrosome (Figures 1C, 2, and 3B; Movies S1, S3, S4, and S5). These data support the idea that the docked centrosome organizes the microtubule network so secretory lysosomes, Golgi stacks, and recycling endosomes can be readily focused at the plasma membrane by minus end-directed transport toward the centrosome.

Small bumps and membrane projections have been described at the site of centrosome polarization during target cell killing [2, 5]. Tomographic analysis showed five out of nine docked mother centrioles were enclosed in a membrane bump or protrusion at the surface (Figure 3; Movies S4, S5, and S6). Rotation of the reconstructed tomograms revealed mother centriole distal appendages in contact with the membrane around the circumference of the bump (Figure 3Bd). In some cases, fine filaments were visible between the ends of the distal appendages and the membrane (Figure 3B, arrowheads).

The arrangement of docked mother centrioles in CTLs showed similarities to that reported for basal bodies at the base of cilia below the axoneme. However, unlike basal bodies, longitudinal profiles revealed the distal ends of the CTL mother centrioles were spatially separated from the membrane (Figure 3A; Movie S4). A clear distal end terminus could be seen (Figures 3Bc and 3Bd; Movies S5 and S6), and there was no evidence of extension of distal-end microtubules into an axoneme, or of Y-fibers or other structures indicative of a ciliary transition zone. Thus, the organization of docking centrosomes in CTLs resembles basal bodies at early, but not later, stages of ciliogenesis.

**Figure 4:**
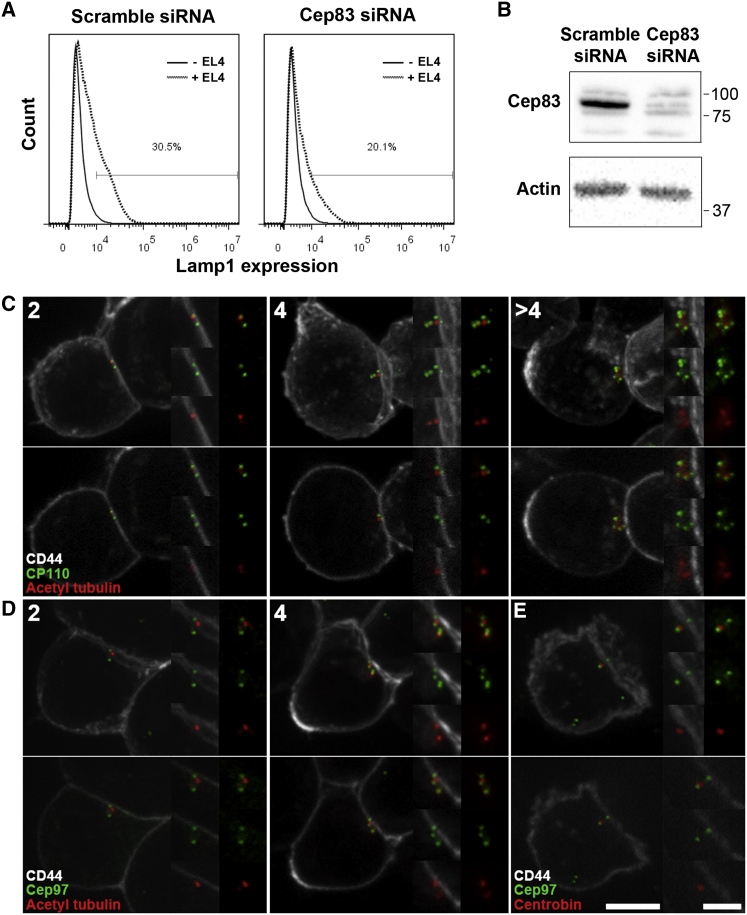
CTLs Require the Distal Appendage Protein Cep83 for Killing and Retain Cep97 and CP110 on Docking (A) Degranulation assay showing Lamp1 appearance on the plasma membrane in OT-I CTLs treated with control (scramble) or Cep83 siRNA, before (–EL4) or after (+EL4) encounter with target cells. (B) Western blots showing Cep83 and actin upon Cep83 siRNA treatment. (C–E) Confocal projection (top) and single-section (bottom) images of synapses with centrosomes at the plasma membrane of OT-I (C and D) or C57 CTLs (E) showing CP110 (green; C) and Cep97 (green; D and E) are retained on docked centrioles (acetylated tubulin, red; C and D) in CTLs with two, four, or more than four CP110/Cep97-positive structures, including the docked mother centrioles that lack the daughter centriole marker centrobin (red; E). Scale bars, 5 μm and 2.5 μm (insets). See also Figure S2.

Recent studies have revealed a hierarchy of distal appendage proteins required for ciliogenesis [11, 21] identifying a key role for Cep83 in centriole docking [11]. We asked whether Cep83 was required for CTL secretion by targeting Cep83 with small interfering RNA (siRNA) and measuring the appearance of the lysosomal membrane protein Lamp1 (CD107a) on the plasma membrane [22] (Figures 4A and 4B). Cep83 depletion reduced the population of CTLs secreting in response to targets from 30% to 20%. These results reveal a role for Cep83 in CTL exocytosis and support the idea that centrosome docking via the mother centriole distal appendages is important for CTL secretion.

Given the striking similarities with ciliogenesis, we were curious to know why a cilium does not form at the immunological synapse. During ciliogenesis the regulatory protein CP110 and its binding partner Cep97 dissociate from the distal end of the mother centriole. This dissociation is required for axoneme growth and cilia formation on membrane docking [12], and CP110 and Cep97 localize only to the daughter centriole in ciliated cells. Since our studies on centriole number had shown CTLs express CP110 (Figure S1), we asked how CP110 behaved during docking of the centrosome at the synapse.

CTL-target cell conjugates were labeled with antibodies against CP110 and acetylated tubulin or centrin-3 to identify centrioles, and the number of CP110-positive structures was assessed in cells with centrioles in contact with the plasma membrane markers CD44, CD8, or PKC-θ (Figures 4C and S2). As reported for other cell types [17, 18], CP110 labeled the ends of the centriole barrels in CTLs, regardless of centriole number. Quantitative analysis of CP110 in 325 CTL conjugates revealed only even numbers of CP110-labeled structures within CTLs at all stages of polarization, including all 55 conjugates with docked centrioles in contact with the membrane. CP110 labeling appeared closer than centrin-3 to the plasma membrane (marked by CD8 or PKC-θ) in 95% CTLs with docked centrosomes (n = 55), indicating that CP110 was not lost from centrioles on association with the plasma membrane (Figure S2). The CP110 binding partner Cep97 also remained associated with mother centrioles docked at the plasma membrane, marked by CD44 (Figure 4D). Docked centrioles retaining Cep97 lacked the daughter centriole marker, centrobin [23], identifying them as mother centrioles (Figure 4E). These results suggest that the CP110/Cep97 complex is not lost from the CTL mother centriole upon membrane docking at the synapse, thus contrasting with the dissociation of these proteins from basal bodies during ciliogenesis.

Taken together, our data show CTLs can polarize the centrosome without exiting the cell cycle. We find CTL mother centrioles dock at the synapse membrane via distal appendages in a manner analogous to mother centriole docking during ciliogenesis but fail to progress to axoneme and transition zone formation. The retention of CP110/Cep97 upon docking is consistent with a block in further ciliogenesis. Interestingly, a recent report showed cilia formation can be induced by CP110 depletion and serum starvation in immune-derived cell lines, suggesting that immune cells have the capacity for ciliogenesis [24]. CP110 levels vary with cell-cycle state with low levels present in quiescent cells and high levels in proliferating cells. It is thought this could prevent aberrant cilia formation during cell division [12, 25]. This raises the possibility that a similar regulation of CP110 in proliferating CTLs might occur, favoring a transient contact of the centrosome with the immunological synapse, allowing CTLs to rapidly repolarize the centrosome and kill multiple targets sequentially.

It has been suggested that polarized release of secretory lysosomes from CTLs may not require the centrosome to contact the membrane [9] but might use short plus-end-directed microtubule motors to reach the synapse [10]. Here, we provide a 3D tomographic analysis showing that the centrosome docks directly at the immunological synapse via the distal appendages of the mother centriole. This organizes the microtubule network directly from the plasma membrane allowing secretory lysosomes to be delivered to the immunological synapse via a minus-end-directed motor alone [1, 4].

Our results not only reveal striking similarities between centrosome docking at the immunological synapse and during early ciliogenesis, but also provide both possible mechanistic and functional explanations as to why cilium formation does not proceed past centrosome docking at the synapse in cytolytic cells.

## Experimental Procedures

### Cell Preparation and Culture

Breeding of genetically modified mice used in this study were licensed by the appropriate UK regulatory authority (Animals, Scientific Procedures Act, 1986), under project license PPL 80/2415, and were approved by the local ethics committee. CTLs from C57BL/6, OT1, and F5 mice, and P815 and EL4 target cells were cultured as in the Supplemental Experimental Procedures or described previously [26, 27].

### TEM and Tomography

CTLs were prepared for TEM as described previously [26, 27]. Thin (50–70 nm) and semi-thin (100–150 nm) sections were used for conventional TEM and thick (400–500 nm) sections used for tomography (see the Supplemental Experimental Procedures).

### Immunofluorescence Microscopy

CTLs and targets were conjugated and adhered to slides at 37°C for 20–30 min, processed, and imaged as in the Supplemental Experimental Procedures. Primary antibodies used were rabbit anti-Cep164 (Novus Biologicals), CP110 or Cep97 (Proteintech); rat anti-CD44 (BD Transduction Labs) or CD8 (YTS192; H Waldmann, Oxford University); and mouse anti-centrobin (Abcam), acetylated tubulin or gamma-tubulin (Sigma-Aldrich), centrin-3 (Novus Biologicals), Lck (Merck Millipore), or PKC-θ (BD Transduction Labs).

### RNAi, Degranulation, and Western Blotting

Non-targeting siRNA and murine Cep83 siRNA were obtained from Dharmacon (ON-TARGET plus Smart pool L-063954 and L-045514). 5 × 10^6^ CTLs were nucleofected with 3 μg of siRNA using Mouse T Nucleofector Kit (Amaxa) and X-001 program according to manufacturer’s instructions. Knockdown efficiency and degranulation were assessed 24 hr post-nucleofection as described in the Supplemental Experimental Procedures.

## Author Contributions

J.C.S. and G.M.G. designed the study and wrote the paper. J.C.S. carried out all imaging and analysis, with help from J.M.M. and P.V. for tomography acquisition. L.O.R. and K.L.A. carried out siRNA experiments.

## Supplemental Information

Document S1. Supplemental Experimental Procedures and Figures S1 and S2

Movie S1. CTLs with Multiple Centriole Pairs Polarize Their Centrosomes to the Surface on Interaction with Targets, Related to Figure 1 Video file showing the full tomogram reconstruction corresponding to data shown in Figure 1C.

Movie S2. CTL Centrosomes Adopt the Topology of Basal Bodies and Reorganize Microtubule Networks to Pass under the Contact Membrane, Related to Figure 2 Video file showing the full tomogram reconstruction corresponding to data shown in Figure 2A.

Movie S3. CTL Mother Centrioles Associate with the Membrane via Their Distal Appendages, Related to Figure 2 Video file showing the full tomogram reconstruction corresponding to data shown in Figure 2B.

Movie S4. The Morphology of Docked CTL Centrosomes at the Membrane Resembles Ciliary Basal Bodies at Early, but Not Late, Stages of Ciliogenesis, Related to Figure 3 Video file containing the full tomogram reconstruction corresponding to data shown in Figure 3A.

Movie S5. The Morphology of Docked CTL Centrosomes at the Membrane Resembles Basal Bodies at Early, but Not Late, Stages of Ciliogenesis, Related to Figure 3 Video file containing the full tomogram reconstruction corresponding to image data shown in Figure 3Ba.

Movie S6. The Morphology of Docked CTL Centrosomes at the Membrane Resembles Basal Bodies at Early, but Not Late, Stages of Ciliogenesis, Related to Figure 3 Video file containing the cropped area of the tomogram shown in Movie S5, containing the docked centrosome only and corresponding to data shown in Figures 3Bb and 3Bc.

Document S2. Article plus Supplemental Information
