# Supplementary material for: Mother Centriole Distal Appendages Mediate Centrosome Docking at the Immunological Synapse and Reveal Mechanistic Parallels with Ciliogenesis
Source: Curr Biol. 2015 Dec 21;25(24):3239–44. doi: 10.1016/j.cub.2015.10.028 (PMC4691242; doi:10.1016/j.cub.2015.10.028)
Supplement: Document S1. Supplemental Experimental Procedures and Figures S1 and S2 [file mmc1.pdf]

Current Biology

Supplemental Information

Mother Centriole Distal Appendages Mediate Centrosome Docking at the Immunological Synapse and Reveal Mechanistic Parallels with Ciliogenesis

Jane C. Stinchcombe, Lyra O. Randzavola, Karen L. Angus, Judith M. Mantell, Paul Verkade, and Gillian M. Griffiths

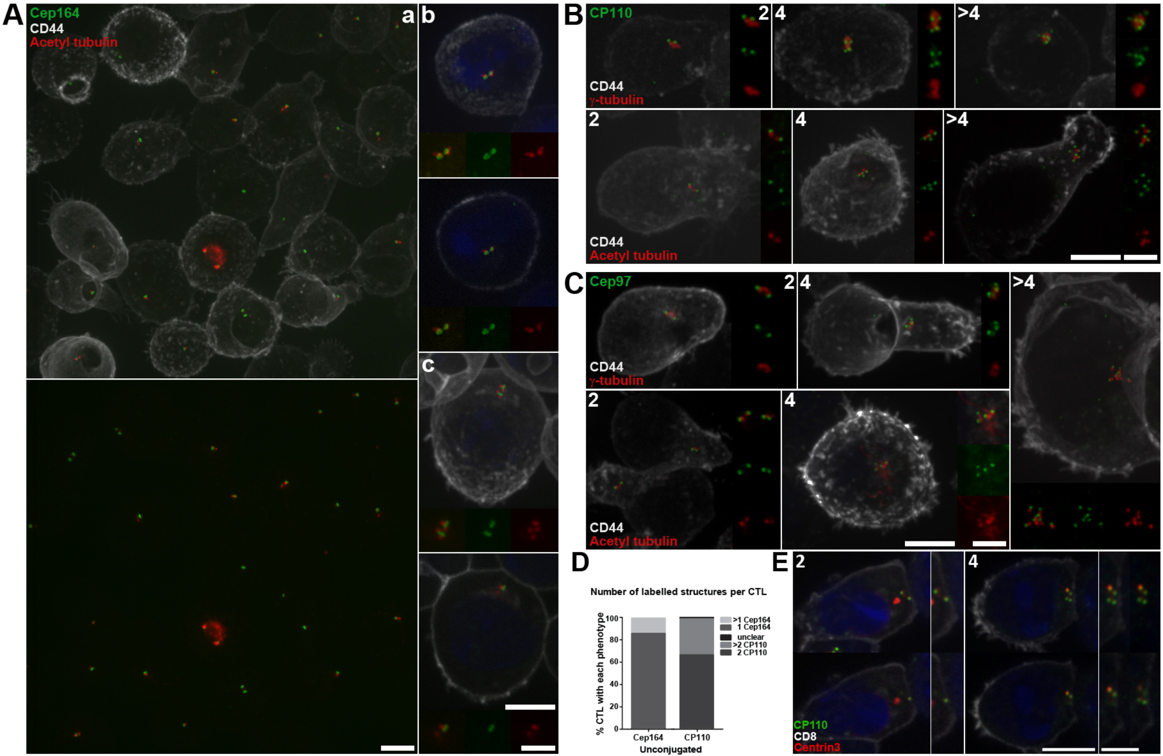

A
Cep164
CD44
Acetyl tubulin
a
b
c
B
CP110
CD44
γ-tubulin
2
4
>4
CD44
Acetyl tubulin
C
Cep97
CD44
γ-tubulin
CD44
Acetyl tubulin
D
Number of labelled structures per CTL
% CTL with each phenotype
>1 Cep164
1 Cep164
unclear
>2 CP110
2 CP110
Cep164
CP110
Unconjugated
E
CP110
CD8
Centrin3

Figure S1

Figure S1, related to Figure 1. CTL can contain centrosomes with more than one Cep164-positive mother centriole and/or multiple CP110/Cep97 complexes

A. CTL can contain centrosomes with more than one Cep164-positive mother centriole. Overview (a) and single cell (b-c) confocal images of unconjugated OT-I (a, c) or C57BL/6 (b) CTL labeled with acetylated tubulin (red) for centrioles, the mother distal appendage marker Cep164 (green), and CD44 (white) to delineate the membrane. Multiple-plane projections show total cellular centrosome components (a; top panels b-c) and single plane images show details of the Cep164 labelling (bottom panels b-c). (a) CTL populations contain individual cells with more than one Cep164-positive structure as well as those containing just one. (b-c) Higher power images of the centrioles (insets) show Cep164 is associated with rings characteristic of distal appendage labelling regardless of the number of structures, confirming the presence of multiple mother centrioles in these cells. Nuclei in (b) are stained with Hoechst, blue.

B-C. CTL can contain 2, 4 or >4 CP110/Cep97 complexes. Confocal multiple-plane projections images through the region of OT-I (B top row and first panel bottom row; C) or C57BL/6 (B second and third panels bottom row) CTL containing the centrosome, labeled with anti-CP110 (green, B) or Cep97 (green, C), plus CD44 (white) for the surface membrane and either gamma-tubulin for whole centrosomes (red, top rows B and C) or acetylated tubulin for individual centrioles (red, bottom rows B and C). Insets show higher power magnifications of the centriolar region. CP110 (B) and Cep97 (C) have similar distributions in CTL. Cells with 2, 4 or >4 centrosome-associated CP110 (B) or Cep97 (C) positive structures, as indicated, are shown.

D-E. Analysis of the number of Cep164 and/or CP110-positive structures in CTL. D. Quantitation of the number of Cep164 or CP110-positive structures/cell using the centriole barrel protein centrin-3 to identify centrioles and CTL-specific synapse membrane markers (CD8, Lck or PKC-theta) to label the CTL cell membrane. **E.** Illustrative images of CTL-target conjugate samples used for quantitation analyses, labeled with CP110 (green), centrin-3 (red) and CD8 (white), and illustrating polarization of 2 and 4 centrioles to the synapse.

Bars: 5 μm, 2.5 μm (insets)

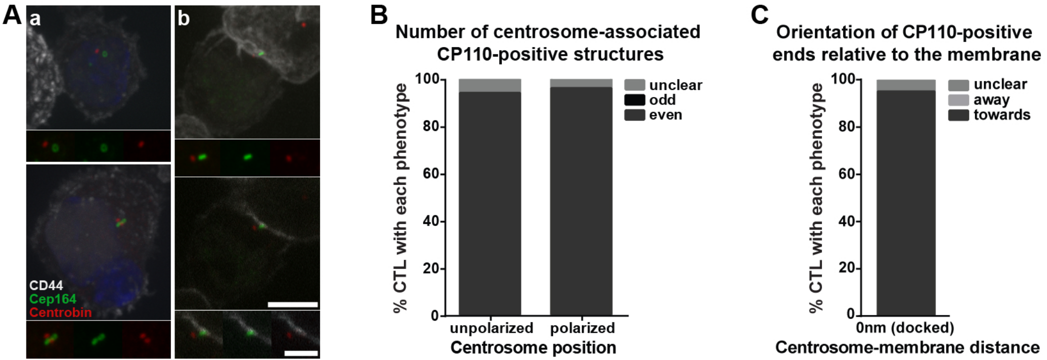
A
a
b
CD44
Cep164
Centrobin
B
Number of centrosome-associated CP110-positive structures
% CTL with each phenotype
100
80
60
40
20
0
unclear
odd
even
unpolarized
polarized
Centrosome position
C
Orientation of CP110-positive ends relative to the membrane
% CTL with each phenotype
100
80
60
40
20
0
unclear
away
towards
0nm (docked)
Centrosome-membrane distance

Figure S2

Figure S2, related to Figures 1 and 4. The CP110/Cep97 complex remains associated with the mother centriole on docking

A. Confocal images of OT-1 CTL alone (a, multiple plane projections) or conjugated to EL4 targets (b, multiple plane projection (top) and single plane through the synapse (bottom)) labeled with antibodies against the daughter centriole marker centrobin (red), the mother centriole marker Cep164 (green) and CD44 for CTL and EL4 membranes (white). Higher power images of the centrioles are shown in insets. (a) Centrobin and Cep164 recognise distinct centrioles in cells with one (a, top) and two (a, bottom) centrosomes. (b) The centrobin-positive daughter centriole lies behind the Cep164-positive mother centriole when Cep164 is associated with the contact site membrane (b). Bars: 5 μm; 2.5 μm (insets)

B-C. Quantitation of samples of CTL-target conjugates prepared for immunofluorescence and quantitation as described for Figures S1D-E and confirming there is no loss of CP110 from CTL centrosomes during killing. (B) The number of CP110 positive structures in polarized versus non polarized cells. (C) The orientation of the CP110-positive distal ends of centrioles at the synapse (defined as CP110-positive end ‘towards’ or ‘away’ from membrane markers on docking).

Supplemental Experimental Procedures

Preparation and culture of CTL from C57BL/6 mice and P815 target cells
CTL isolated from C57BL/6 mice were prepared from splenocytes, and stimulated in CTL medium (RPMI 1640, 10% FCS, 1% L-glutamine, 1% sodium pyruvate, 50 µM β-mercaptoethanol, 100 U/ml IL-2 (Roche) and 50 U/mL penicillin/streptomycin (Gibco)) with equal numbers of irradiated (3000 Rad) Balb/c splenocytes at 37°C. CTL were purified over Ficoll Histopaque-1083-1 (Sigma-Aldrich) after 5-6 days and washed before further culture in CTL medium. P815 mouse target cells were maintained in RPMI, 10% FCS and L-glutamine.

Conjugation of CTL for TEM and TEM tomography
For TEM analysis, CTL were incubated overnight (15–16 hr) in the presence of 1 mg/ml horseradish peroxidase (Serva), washed and resuspended in serum-free RPMI 1640 at 1-2 x 10^6 cells/ml. Labelled CTL were either used unconjugated or mixed 1:1 with P815 target cells and incubated at 37°C for 20-60 min to form conjugates. Samples were fixed in 2% paraformaldehyde with either 1.5% or 3% gluteraldehyde, then processed for diaminobenzidine cytochemistry, osmium fixation, urynal acetate staining en bloc and embedding in EPON as previously described [S1-4]. Thin and semi-thin sections were viewed using a Phillips C100 TEM (FEI) at magnifications of 6-11 K. Conventional 2D images were captured on Kodak photographic negative film (Kodak) and the negatives used to generate digital electron micrographs using a Flextight X5 scanner (Hasselblad).

TEM tomography
Thick (400-500 nm) sections of CTL conjugated to targets as above were collected on film-coated slot grids, either left unstained or stained with lead citrate, and the top and bottom of each section labeled with 15 nm gold (British Biocell International) for 5 min. Labeled samples were pre-screened using a Phillips C100 TEM to identify cells of interest. Single or dual axis tilt series were collected using an FEI Technai 20, 200 kV TEM equipped with a dual-axis holder (FEI, Eindhoven) with images taken every 1-1.5 degrees from between -70 and -60 degrees to between +60 and +70 degrees at magnifications of between to 15-29 K using the FEI tomography acquisition software (Xplore3D). Tilt series were processed to generate tomographic

reconstructions using the IMOD and 3dMOD packages (Boulder Laboratory for 3-D EM of cells, University of Colorado, USA). Image J software was used for additional image processing and movie generation.

Images shown in Figure 1C represent low (a-b) and higher power (c) projections of multiple (120, a) or pairs of (b-c) sequential planes taken from the reconstructed tomogram in the regions containing the two mother centrioles (a-b, c-bottom) and a procentriole associated with the trailing mother centriole (c-top). Figure 2A shows projections of multiple (190 (a), 50 (b) or 35 (c)) or pairs of (d) sequential planes through different regions of the polarized centrosome. Images in 2B show projections (55 planes, a) or single, non-sequential planes (b-d) from the tomogram series. For Figure 3 each reconstruction is shown as a series of 'thick' (400-500 nm) (Aa, Ba-b) and 'thin' (50-70 nm) (Ab, Bc-d) section images passing through the whole centriole, generated from projections of multiple consecutive planes (50-60 for Aa i-ii and Aa iv-v; 45, 60, 40 or 70 for Ba i, Ba ii, Bb i, Bb ii, respectively) or pairs of consecutive planes (Ab, Bc-d), and organised sequentially. In (A) the series passes left to right from the cartwheel-bearing proximal end of a procentriole (A i, white arrowhead) transversely through the centriole barrel to the other side (A v). For (B), the series passes from the distal side (Bc i-left, Bd i) to the proximal end (Bc ii-right, d vi). (Aa iii) shows a multiple plane projection image through the whole centriole (200 planes). (A) and (Ba-c) are taken from reconstructions shown in made in the xy plane through the z axis. For (B) where the centriole barrel is tilted to the section plane, reconstructions were also made with the x and y axes tilted to align the z-axis with the axis of the centriole barrel (Bd).

Immunofluorescence and quantitation

OT-I or C57BL/6 CTL alone or conjugated to EL4 or P815 targets respectively, were either fixed with 4% PFA in PBS, quenched with 15 mM glycine in PBS (20 min) and permeabilized with 0.05-0.2% Triton-X100 (5 min) at RT or fixed and permeabilized with -20°C-cooled methanol for 5 min. Samples were blocked in 1-2% BSA in PBS (>15 min) before labelling with primary antibodies (1 hr at RT or overnight at 4°C), followed by goat or donkey anti-mouse, rat or rabbit Alexa-labeled secondary antibodies (Invitrogen or Jackson Stratech) (1 hr at RT). Samples were washed extensively between each processing step. Nuclei were stained with Hoechst

(1:25,000) in PBS for 5 min and slides mounted with Mowiol or Vectashield (Vector Laboratories Inc.). Samples were analysed using an Andor spinning-disk confocal system (Revolution; Andor) fitted with a spinning-disk unit (CSU-X1; Yokogawa) and Olympus IX81 microscope, and viewed using Olympus 100x and 60x objectives with numerical apertures of 1.40 and 1.42, respectively, and lasers exciting at 405, 488, 543 and 633 nm. Z-stack image series were collected across the depth of the cell containing the total centrosomal content at 0.1 µm intervals and captured using a 512 × 512, 16 μm^2-pixel camera (iXon; Andor) using IQ software (Andor). Image series were analyzed using Imaris (Bitplane, Switzerland) software to create single plane images and/or projection images of 3D reconstructions and processed with Adobe Photoshop (CS4-CS6; Adobe) software.)

Quantitation of immunofluorescence samples

A total of 317 CP110- and 347 Cep164-labeled unconjugated CTL, and 435 CP110- and 320 Cep164-labeled CTL conjugated to targets were quantitated. Samples were co-labeled with primary antibodies against centrin-3, CD8, Lck or PKC-theta and analysed using 3D reconstructions of Z-stack series created using Imaris software. Centrosomes were defined as centrin-3 barrels plus CP110 or Cep164 labeling. Polarization of the centrosome was determined by measuring the shortest distance between the centriole nearest the membrane and the synapse plasma membrane. Orientation of centrioles at the synapse was defined by the position of the CP110-labeled distal end of centrin-3 positive centriole barrels relative to the plasma membrane markers (i.e. end facing ‘towards’ or ‘away’ from the membrane). Orientation of the mother centriole was given by the position (towards or away) of the Cep164-labeled distal end of the Cep164-positive centrin-3-labeled centriole relative to the membrane.

Degranulation assay and western blotting of siRNA nucleofected cells

For degranulation assay, CTLs nucleofected with either scramble siRNA or Cep83 siRNA were mixed at 1:1 ratio with EL-4 target cells in 96-well plate at 37°C in the presence of CD107a-PE (LAMP1). After 3 hr of incubation, cells were harvested into cold PBS and resuspended in PBS/0.2% FBS (FACS buffer). Anti CD8-APC was used to differentiate effector CTL and percent of degranulation was measured by analysing Lamp1 expression [S4].

For western blotting CTL were lysed at 2 x 10^7 cells/ml in lysis buffer (150 mM NaCl, 50 mM Tris-HCl pH 8, 1 mM MgCl2, 2% Triton X-100) supplemented with Protease Inhibitor Cocktail (Roche). Samples were loaded in 2x Laemmli Buffer on 4-12% NuPage Bis-Tris Gel (Invitrogen) under reducing conditions in MES Buffer (Formedium), transferred to nitrocellulose membrane (Amersham) using NuPage transfer buffer (Invitrogen) and incubated in TBS, 5% milk, 0.05% Tween-20 (Sigma) with rabbit anti-Cep83 and mouse anti-actin (Sigma) primary antibodies followed by anti-rabbit IgG-horseradish peroxidase and anti-mouse IgG-horseradish peroxidase (Thermo Fisher Scientific) secondary antibodies. Blots were developed with ECL Prime reagent (Amersham) and signal was recorded on a ChemiDoc MP Imager (Biorad).

Supplemental References

S1. Stinchcombe, J.C., Salio, M., Cerundolo, V., Pende, D., Arico, M., and Griffiths, G.M. (2011). Centriole polarisation to the immunological synapse directs secretion from cytolytic cells of both the innate and adaptive immune systems. BMC Biology *9*, 45.

S2. Stinchcombe, J.C., Barral, D.C., Mules, E.H., Booth, S., Hume, A.N., Machesky, L.M., Seabra, M.C., and Griffiths, G.M. (2001). Rab27a is required for regulated secretion in cytotoxic T lymphocytes. The Journal of Cell Biology *152*, 825-834.

S3. Stinchcombe, J.C., Bossi, G., Booth, S., and Griffiths, G.M. (2001). The immunological synapse of CTL contains a secretory domain and membrane bridges. Immunity *15*, 751-761.

S4. Jenkins, M.R., Tsun, A., Stinchcombe, J.C., and Griffiths, G.M. (2009). The strength of T cell receptor signal controls the polarization of cytotoxic machinery to the immunological synapse. Immunity *31*, 621-631.
