# Supplementary figures and images for: Mother Centriole Distal Appendages Mediate Centrosome Docking at the Immunological Synapse and Reveal Mechanistic Parallels with Ciliogenesis
Source: Curr Biol. 2015 Dec 21;25(24):3239–44. doi: 10.1016/j.cub.2015.10.028 (PMC4691242; doi:10.1016/j.cub.2015.10.028)

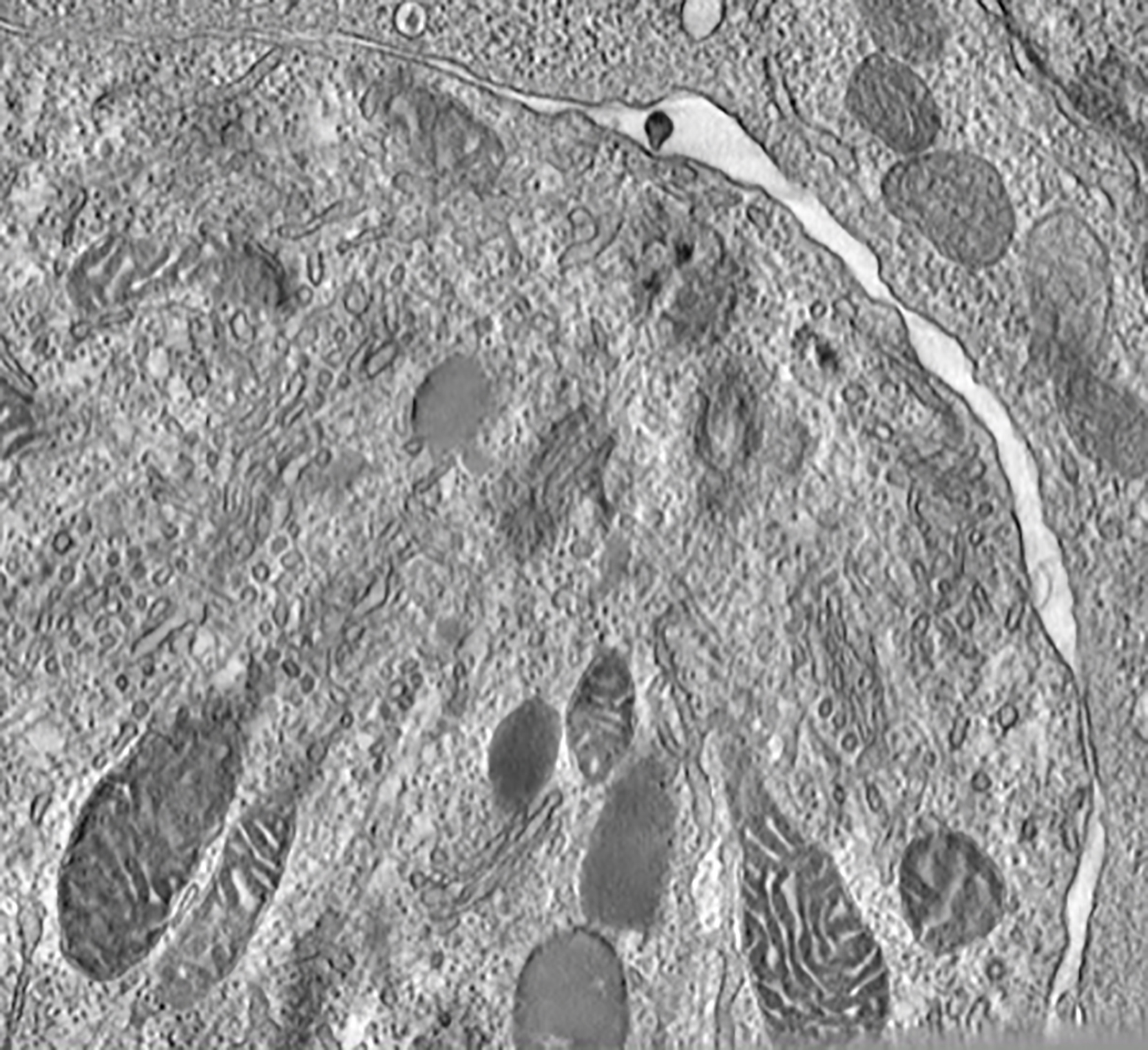

Supplement: Movie S1. CTLs with Multiple Centriole Pairs Polarize Their Centrosomes to the Surface on Interaction with Targets, Related to Figure 1 — Video file showing the full tomogram reconstruction corresponding to data shown in Figure 1C. [file mmc2.jpg]

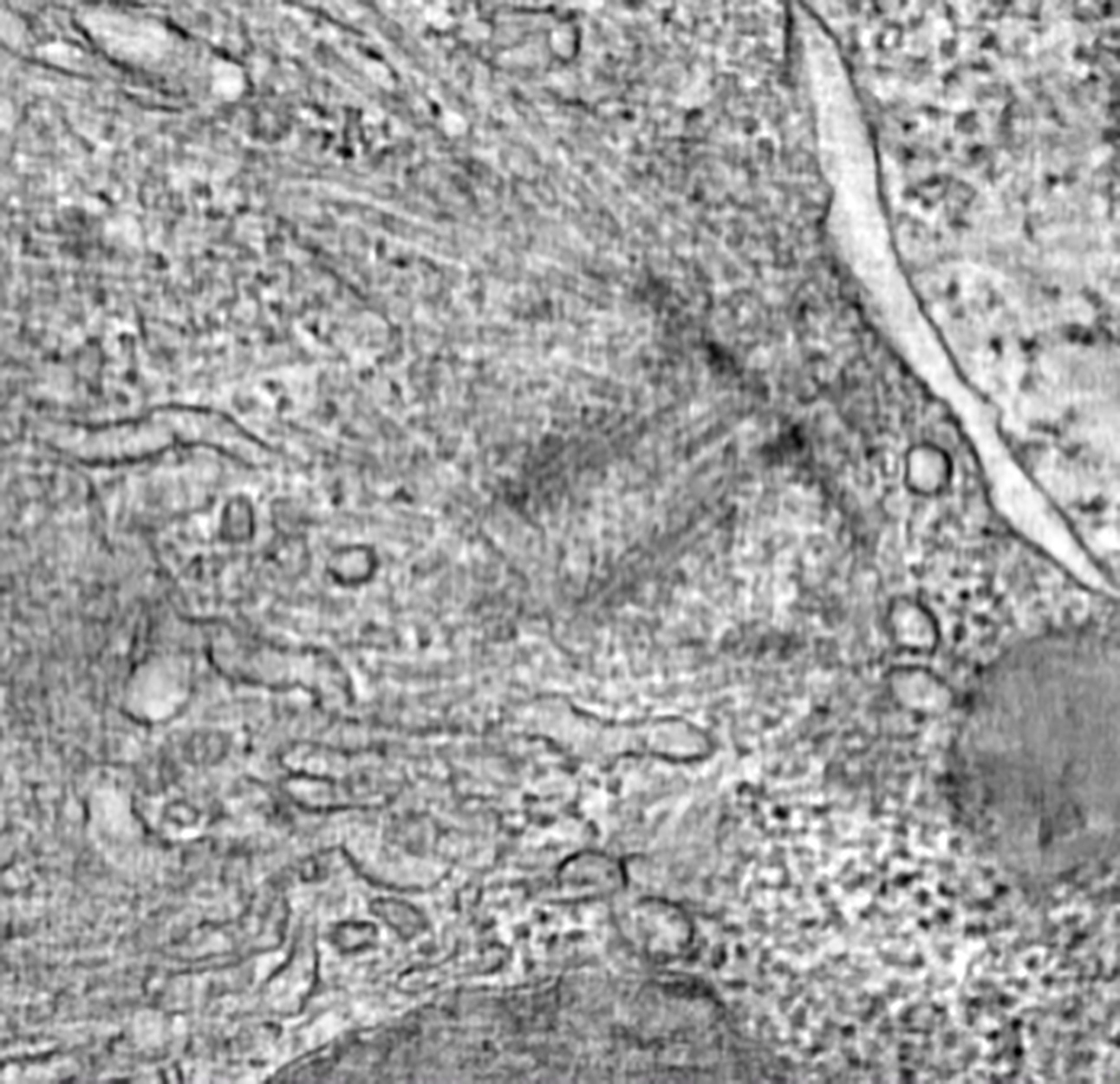

Supplement: Movie S2. CTL Centrosomes Adopt the Topology of Basal Bodies and Reorganize Microtubule Networks to Pass under the Contact Membrane, Related to Figure 2 — Video file showing the full tomogram reconstruction corresponding to data shown in Figure 2A. [file mmc3.jpg]

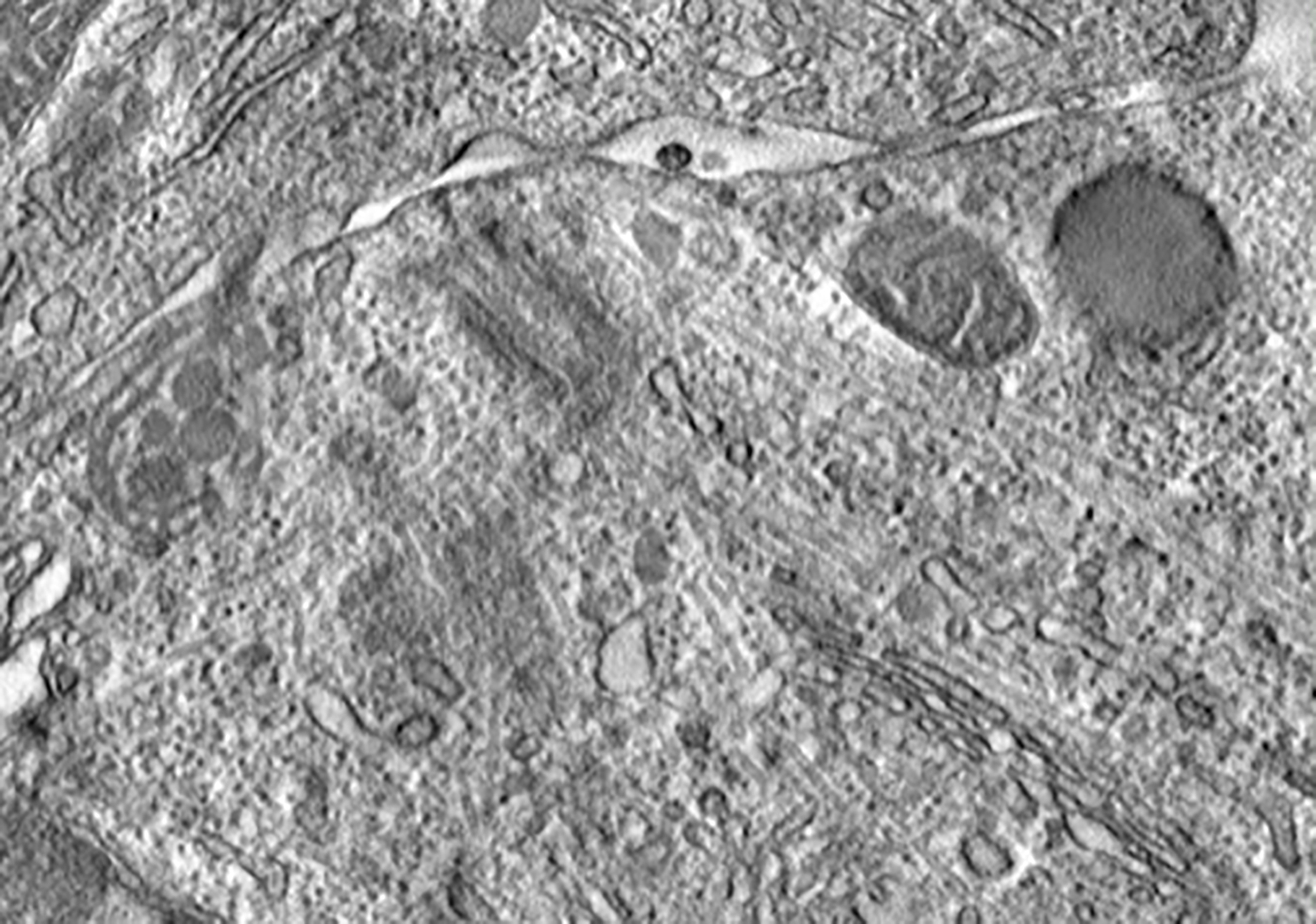

Supplement: Movie S3. CTL Mother Centrioles Associate with the Membrane via Their Distal Appendages, Related to Figure 2 — Video file showing the full tomogram reconstruction corresponding to data shown in Figure 2B. [file mmc4.jpg]

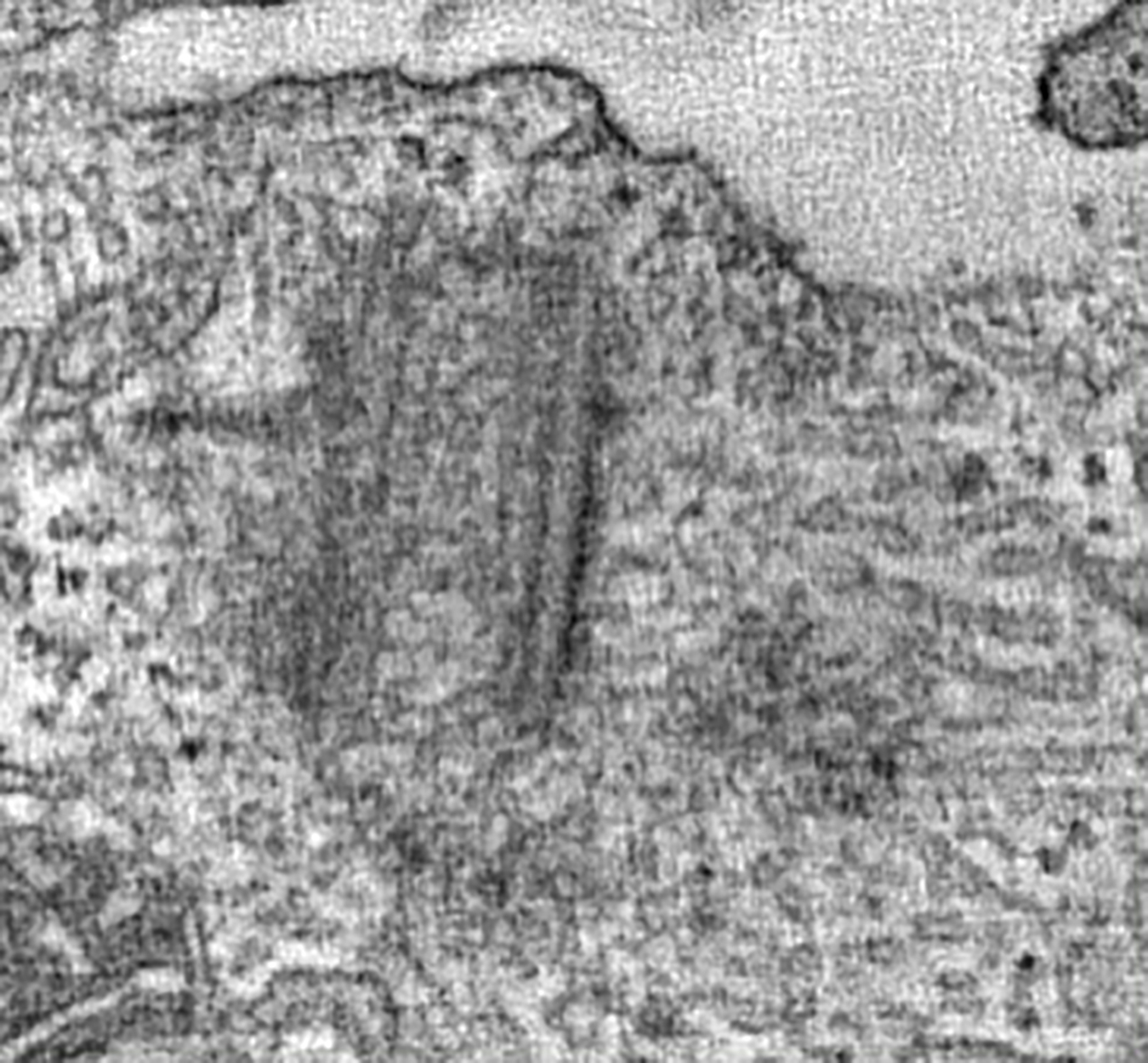

Supplement: Movie S4. The Morphology of Docked CTL Centrosomes at the Membrane Resembles Ciliary Basal Bodies at Early, but Not Late, Stages of Ciliogenesis, Related to Figure 3 — Video file containing the full tomogram reconstruction corresponding to data shown in Figure 3A. [file mmc5.jpg]

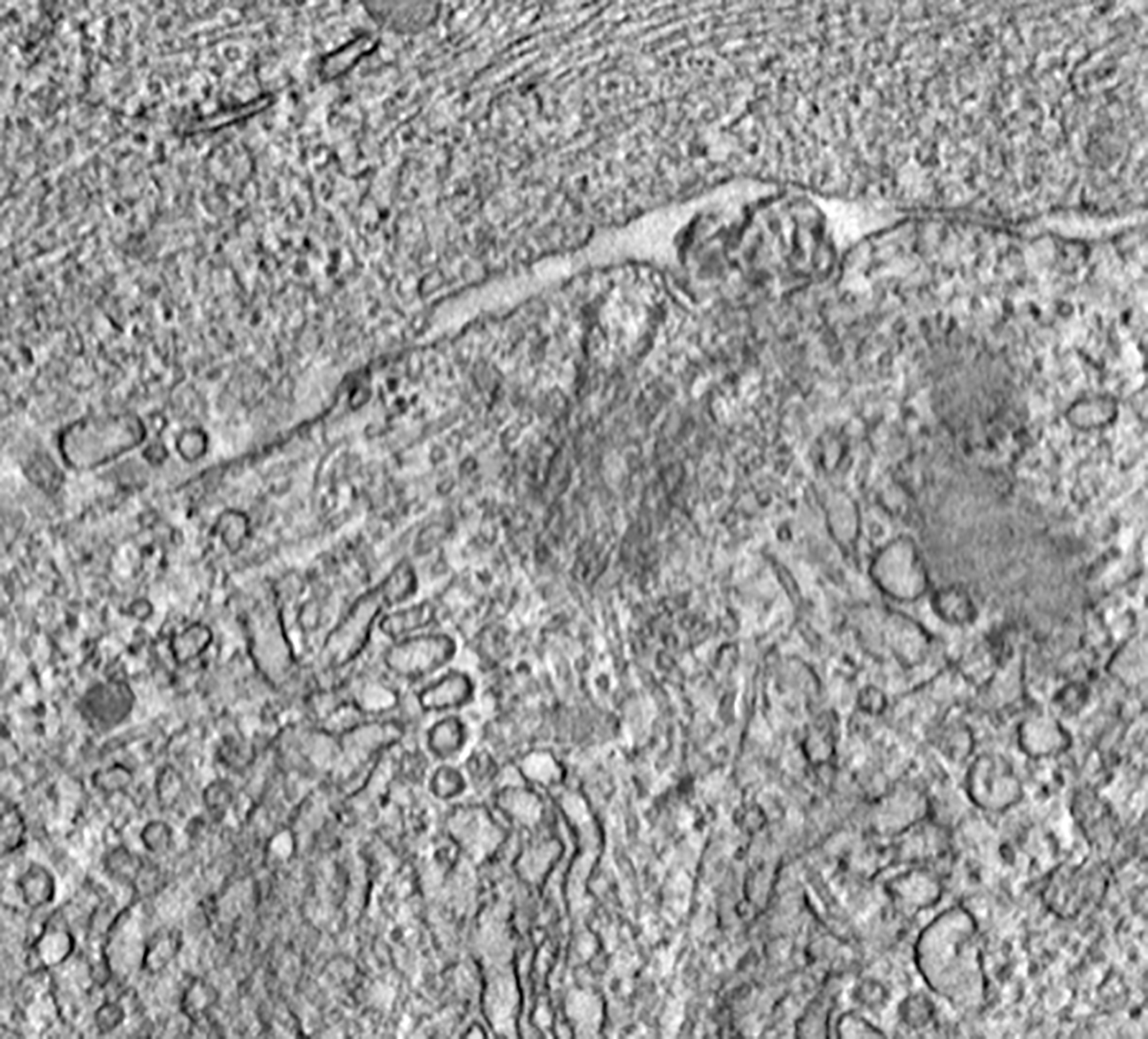

Supplement: Movie S5. The Morphology of Docked CTL Centrosomes at the Membrane Resembles Basal Bodies at Early, but Not Late, Stages of Ciliogenesis, Related to Figure 3 — Video file containing the full tomogram reconstruction corresponding to image data shown in Figure 3Ba. [file mmc6.jpg]

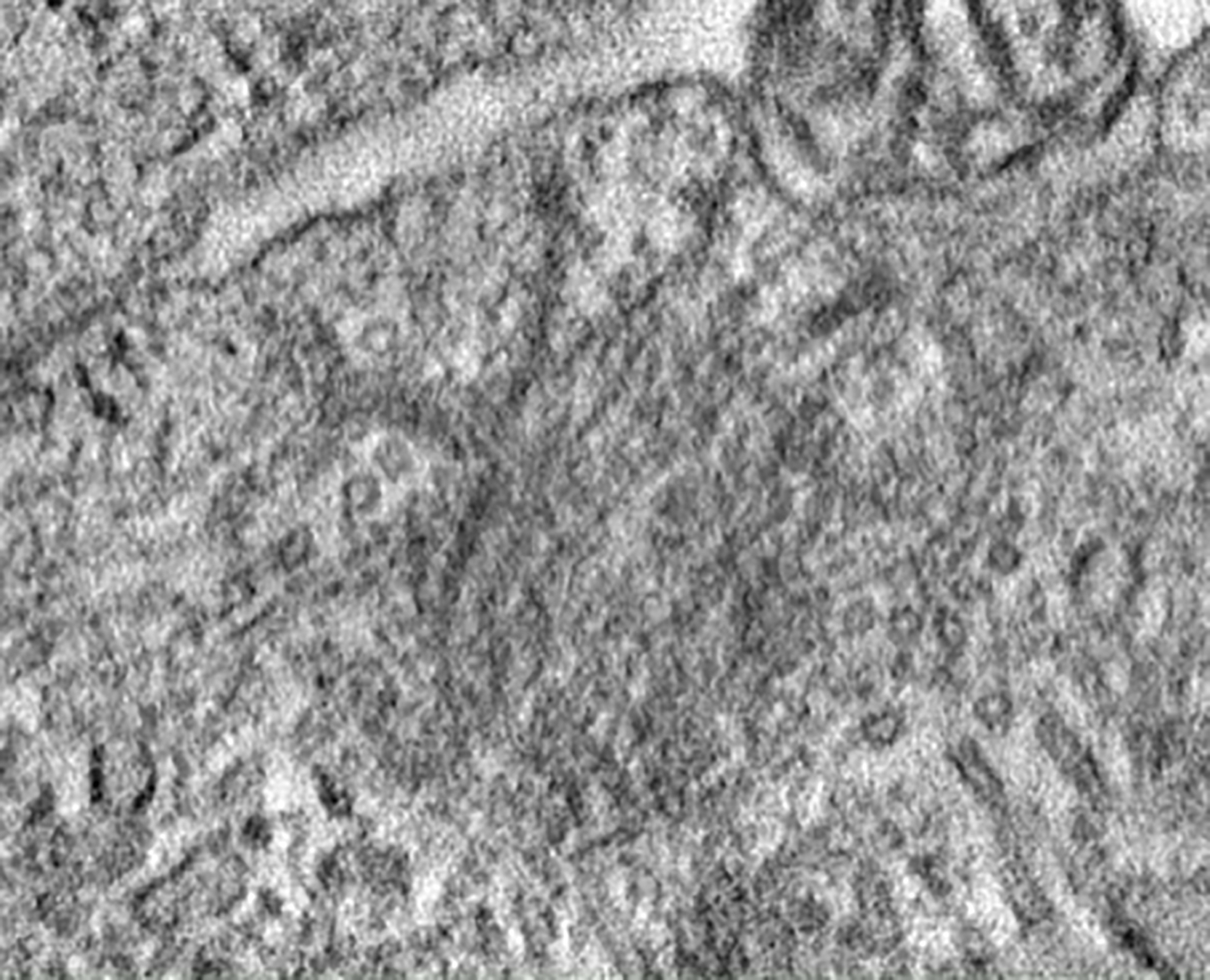

Supplement: Movie S6. The Morphology of Docked CTL Centrosomes at the Membrane Resembles Basal Bodies at Early, but Not Late, Stages of Ciliogenesis, Related to Figure 3 — Video file containing the cropped area of the tomogram shown in Movie S5, containing the docked centrosome only and corresponding to data shown in Figures 3Bb and 3Bc. [file mmc7.jpg]
