# Supplementary material for: Mother Centriole Distal Appendages Mediate Centrosome Docking at the Immunological Synapse and Reveal Mechanistic Parallels with Ciliogenesis
Source: Curr Biol. 2015 Dec 21;25(24):3239–44. doi: 10.1016/j.cub.2015.10.028 (PMC4691242; doi:10.1016/j.cub.2015.10.028)
Supplement: Document S2. Article plus Supplemental Information [file mmc8.pdf]

Report

Current Biology

# Mother Centriole Distal Appendages Mediate Centrosome Docking at the Immunological Synapse and Reveal Mechanistic Parallels with Ciliogenesis

## Graphical Abstract

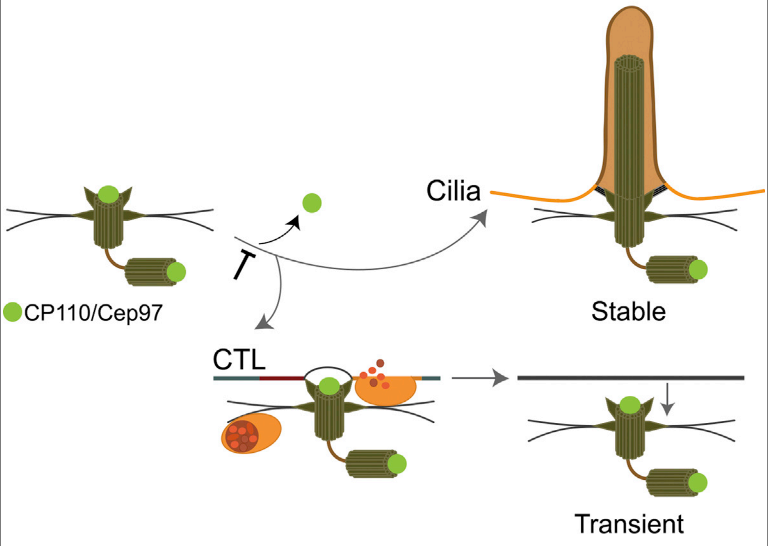


## Highlights

- Centrosome docking at the immune synapse parallels early ciliogenesis
- CTL mother centriole distal appendages dock directly with the membrane
- Cep83 depletion inhibits CTL secretion
- CP110 is retained on the mother centrioles and ciliogenesis is blocked

## Authors

Jane C. Stinchcombe, Lyra O. Randzavola, Karen L. Angus, Judith M. Mantell, Paul Verkade, Gillian M. Griffiths

## Correspondence

gg305@cam.ac.uk

## In Brief

Stinchcombe et al. show that centrosome docking at the immunological synapse of CTLs mirrors early ciliogenesis, with the mother centriole docking via distal appendages, but further ciliogenesis blocked. The authors propose that transient centrosome docking without ciliogenesis will favor the rapid serial killing used by CTLs.

Stinchcombe et al., 2015, Current Biology *25*, 3239–3244
December 21, 2015 ©2015 The Authors
http://dx.doi.org/10.1016/j.cub.2015.10.028

Current Biology
Report

# Mother Centriole Distal Appendages Mediate Centrosome Docking at the Immunological Synapse and Reveal Mechanistic Parallels with Ciliogenesis

Jane C. Stinchcombe,[1] Lyra O. Randzavola,[1] Karen L. Angus,[1] Judith M. Mantell,[2] Paul Verkade,[2] and Gillian M. Griffiths[1,*]
[1]Cambridge Institute for Medical Research, Cambridge Biomedical Campus, Hills Road, Cambridge CB2 0XY, UK
[2]Wolfson Bioimaging Facility, University of Bristol, Medical Sciences, Bristol BS8 1TD, UK
*Correspondence: gg305@cam.ac.uk
http://dx.doi.org/10.1016/j.cub.2015.10.028
This is an open access article under the CC BY license (http://creativecommons.org/licenses/by/4.0/).

## SUMMARY

**Cytotoxic T lymphocytes (CTLs) are highly effective serial killers capable of destroying virally infected and cancerous targets by polarized release from secretory lysosomes. Upon target contact, the CTL centrosome rapidly moves to the immunological synapse, focusing microtubule-directed release at this point [1–3]. Striking similarities have been noted between centrosome polarization at the synapse and basal body docking during ciliogenesis [1, 4–8], suggesting that CTL centrosomes might dock with the plasma membrane during killing, in a manner analogous to primary cilia formation [1, 4]. However, questions remain regarding the extent and function of centrosome polarization at the synapse, and recent reports have challenged its role [9, 10]. Here, we use high-resolution transmission electron microscopy (TEM) tomography analysis to show that, as in ciliogenesis, the distal appendages of the CTL mother centriole contact the plasma membrane directly during synapse formation. This is functionally important as small interfering RNA (siRNA) targeting of the distal appendage protein, Cep83, required for membrane contact during ciliogenesis [11], impairs CTL secretion. Furthermore, the regulatory proteins CP110 and Cep97, which must dissociate from the mother centriole to allow cilia formation [12], remain associated with the mother centriole in CTLs, and neither axoneme nor transition zone ciliary structures form. Moreover, complete centrosome docking can occur in proliferating CTLs with multiple centriole pairs. Thus, in CTLs, centrosomes dock transiently with the membrane, within the cell cycle and without progression into ciliogenesis. We propose that this transient centrosome docking without cilia formation is important for CTLs to deliver rapid, repeated polarized secretion directed by the centrosome.**

## RESULTS AND DISCUSSION

Activated CTLs proliferate rapidly, suggesting that some CTLs may contain replicating centrioles. Although ciliogenesis is often reported to occur during the G0-G1 transition and require exit from the cell cycle [13–15], there are several instances of cilia formation in cycling and differentiated cells [16]. This suggested centrosome polarization in CTLs might continue throughout activated populations, regardless of cell-cycle state. To address this, we investigated whether centrosome docking can occur in proliferating CTLs undergoing centriole duplication.

EM analysis of CTLs (Figures 1A and 1B) revealed the presence of cells with procentriole-bearing centrioles, consistent with replication events (Figure 1B). Curiously, we also observed cells with more than one complete mature centriole pair, indicated by cells with multiple pairs, each with an appendage-bearing mother centriole (Figure 1A). To determine the frequency of CTLs with multiple or replicating centriole pairs, we fixed CTLs and labeled them for immunofluorescence with antibodies against acetylated tubulin, gamma tubulin, or centrin-3 to identify both mother and daughter centrioles and either CP110 or Cep97 to identify the distal ends of all centrioles and procentrioles [12, 17] or Cep164, a mother centriole distal appendage protein [18] (Figures S1 and S2). Using this approach, more than two CP110- or Cep97-positive structures per cell identified multiple centriole pairs and replicating centrioles while more than one Cep164-positive structure identified more than one mature mother centriole. CTLs with two or more Cep164-positive or with two, four, six, eight, or, occasionally, more than eight CP110- or Cep97-positive structures were all observed (Figures S1A–S1C). Quantitation of CP110- (n = 317) or Cep164- (n = 347) labeled CTLs revealed 32% contained more than two CP110-labeled structures, while 14% contained more than one Cep164-labeled ring (Figure S1D). Thus, one-third of CTLs within the population showed centriole duplication and/or more than one pair of centrioles.

As so many CTLs possessed multiple or replicating centrioles, we asked whether cells with replicating, or more than one pair of, centrioles could polarize their centrosome to the synapse during interaction with a target. TEM tomography revealed that CTLs polarize their centrioles to the plasma membrane regardless of centriole number or state of replication (Figure 1C; Movie S1). In CTLs with more than one centriole

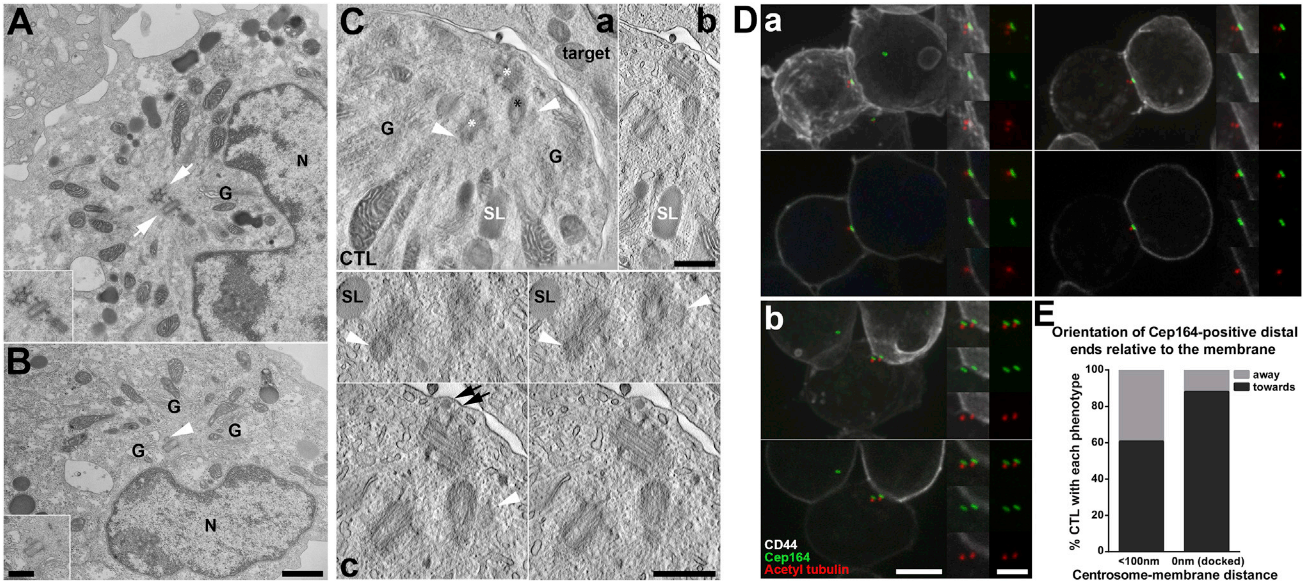


**Figure 1. CTLs Contain Multiple Centriole Pairs, which All Polarize toward the Target with a Mother Centriole nearest the Membrane**

(A and B) 100–200 nm (A) or 50–100 nm (B) TEM sections from C57BL/6 CTLs with more than one appendage-bearing mother centriole (white arrows and inset, A) or centrioles with associated procentrioles (white arrowhead and inset, B). G, Golgi; N, nucleus. Scale bars, 1 μm and 500 nm (insets). See also Figure S1.

(C) Multiple (Ca) or paired (Cb and Cc) plane projection images from a TEM tomograph of an F5 CTL-EL4 target synapse where the CTL contains more than one distal-appendage-bearing mother centriole (white asterisks, Ca) and centrioles bearing procentrioles (white arrowheads, Ca and Cc). Black arrows, fibrous connections between sub-distal appendages and the membrane; SL, secretory lysosome; white SL, secretory lysosome associated with microtubules; G, Golgi. Scale bars, 500 nm. See also Movie S1.

(D) Confocal projection (top) or single-plane (bottom) images of OT-I CTLs (left) conjugated to EL4 targets (right), labeled with acetylated tubulin (centrioles, red), Cep164 (mother centriole distal appendages, green), and CD44 (plasma membrane of both CTLs and EL4 targets, white). CTLs with one (Da) or two (Db) tightly polarized mother centrioles are shown. Scale bars, 5 μm and 2.5 μm (insets).

(E) Quantitation of mother centriole orientation at the CTL membrane in conjugates, assessed by the proximity and direction of Cep164-positive distal ends relative to the membrane.

pair, all centrioles were polarized at the synapse, but only one pair was closely associated with the plasma membrane, with the additional pairs lying just behind the leading pair (Figure 1C; Movie S1).

To determine the frequency at which duplicating centrioles dock, we labeled CTL-target conjugates with antibodies against centrin-3 to identify both mother and daughter centrioles and either CP110 or mother centriole-specific Cep164. 10% Cep164-labeled CTLs (n = 320) contained more than one Cep164-labeled centriole, while 45% CP110-labeled cells (n = 435) had more than two CP110-labeled centrioles. CP110 association did not change upon CTL interaction with targets, with more than two CP110-labeled centrioles in 43% CTLs without targets (n = 110), 44% target-interacting CTLs (centrosome >0.5 μm from synapse, n = 137), and 47% polarized CTLs (centrosome <0.5 μm from synapse; n = 188) (Figure S1E). CTLs with more than one Cep164-labeled centriole increased upon centrosome polarization from 3% in CTLs without targets (n = 76), 7% unpolarized target-conjugated CTLs (n = 89) to 16% polarized conjugated CTLs (n = 155). Although the significance of this increase in polarized cells is not clear, our results show neither replication state nor multiple centrioles prevented polarization of CTL centrioles. Thus, CTLs do not need to exit the cell cycle for centrosome polarization.

Cilia and flagellar basal bodies dock directly at the plasma membrane, with the mother centriole attached via the distal appendages and the daughter centriole behind [19, 20]. We noted that Cep164 and CD44 plasma membrane marker labeling often overlapped (Figure 1D), suggesting that CTL centrosomes might adopt a specific topology at the membrane. Quantitation revealed the Cep164-labeled ends of the mother centriole faced the plasma membrane in 84% of CTL-target conjugates with docked centrosomes (n = 51) (Figure 1E). This suggested that the distal end of the mother centriole might be preferentially orientated toward the membrane when contacting the plasma membrane.

TEM tomography was used to dissect the polarized centrosome topology in 3D at high resolution. Tomograms of docked centrosomes (n = 9) revealed the mother centriole contacted the plasma membrane directly via the ends of the distal appendages (Figure 2, arrowheads; Movies S2 and S3). The orientation of the docked mother centriole organized microtubules emanating from the sub-distal appendages (arrows) to lie directly underneath the synapse membrane (Figure 2A; Movie S2). 3D reconstructions revealed that most of these microtubules were long, extending directly from the docking site back into the cell body (Figures 2A and 2B; Movies S2 and S3). Secretory lysosomes and Golgi cisternae were aligned along microtubules emanating from the polarized centrosome (Figures 1C, 2, and 3B; Movies S1, S3, S4, and S5). These data support the idea that the docked centrosome organizes the microtubule network so secretory lysosomes, Golgi stacks,

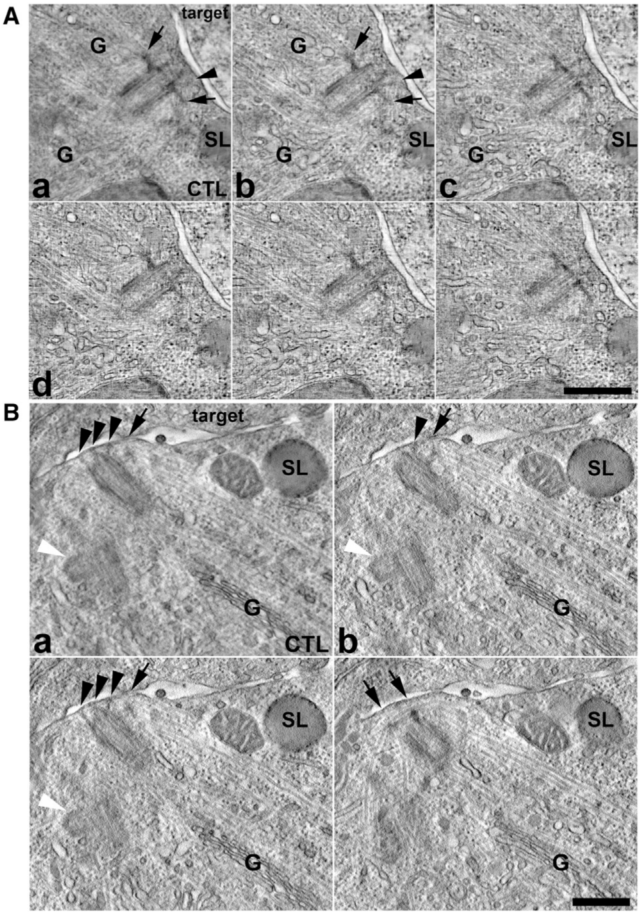


**Figure 2. Centrosome Docking at the CTL Immunological Synapse Resembles Basal Body Docking during Ciliogenesis**

Multiple (Aa–Ac and Ba) or paired (Ad, Bc, and Bd) projection images from dual- (A) or single- (B) axis TEM tomographic reconstructions of synapses formed between F5 CTL (CTL) and EL4 (target) cells revealing (A) the orientation of the polarized mother centriole distal appendages (black arrowheads) and sub-distal appendages (black arrows) at the plasma membrane; (B) CTL mother centrioles dock with the synapse membrane via their distal appendages (black arrowheads), with microtubules radiating out from the sub-distal appendages (black arrows). White arrowheads, procentriole associated with polarized centriole. SL, secretory lysosome; G, Golgi. Scale bars, 500 nm. See also Movies S2 and S3.

and recycling endosomes can be readily focused at the plasma membrane by minus end-directed transport toward the centrosome.

Small bumps and membrane projections have been described at the site of centrosome polarization during target cell killing [2, 5]. Tomographic analysis showed five out of nine docked mother centrioles were enclosed in a membrane bump or protrusion at the surface (Figure 3; Movies S4, S5, and S6). Rotation of the reconstructed tomograms revealed mother centriole distal appendages in contact with the membrane around the circumference of the bump (Figure 3Bd). In some cases, fine filaments were visible between the ends of the distal appendages and the membrane (Figure 3B, arrowheads).

The arrangement of docked mother centrioles in CTLs showed similarities to that reported for basal bodies at the base of cilia below the axoneme. However, unlike basal bodies, longitudinal profiles revealed the distal ends of the CTL mother centrioles were spatially separated from the membrane (Figure 3A; Movie S4). A clear distal end terminus could be seen (Figures 3Bc and 3Bd; Movies S5 and S6), and there was no evidence of extension of distal-end microtubules into an axoneme, or of Y-fibers or other structures indicative of a ciliary transition zone. Thus, the organization of docking centrosomes in CTLs resembles basal bodies at early, but not later, stages of ciliogenesis.

Recent studies have revealed a hierarchy of distal appendage proteins required for ciliogenesis [11, 21] identifying a key role for Cep83 in centriole docking [11]. We asked whether Cep83 was required for CTL secretion by targeting Cep83 with small interfering RNA (siRNA) and measuring the appearance of the lysosomal membrane protein Lamp1 (CD107a) on the plasma membrane [22] (Figures 4A and 4B). Cep83 depletion reduced the population of CTLs secreting in response to targets from 30% to 20%. These results reveal a role for Cep83 in CTL exocytosis and support the idea that centrosome docking via the mother centriole distal appendages is important for CTL secretion.

Given the striking similarities with ciliogenesis, we were curious to know why a cilium does not form at the immunological synapse. During ciliogenesis the regulatory protein CP110 and its binding partner Cep97 dissociate from the distal end of the mother centriole. This dissociation is required for axoneme growth and cilia formation on membrane docking [12], and CP110 and Cep97 localize only to the daughter centriole in ciliated cells. Since our studies on centriole number had shown CTLs express CP110 (Figure S1), we asked how CP110 behaved during docking of the centrosome at the synapse.

CTL-target cell conjugates were labeled with antibodies against CP110 and acetylated tubulin or centrin-3 to identify centrioles, and the number of CP110-positive structures was assessed in cells with centrioles in contact with the plasma membrane markers CD44, CD8, or PKC-$\theta$ (Figures 4C and S2). As reported for other cell types [17, 18], CP110 labeled the ends of the centriole barrels in CTLs, regardless of centriole number. Quantitative analysis of CP110 in 325 CTL conjugates revealed only even numbers of CP110-labeled structures within CTLs at all stages of polarization, including all 55 conjugates with docked centrioles in contact with the membrane. CP110 labeling appeared closer than centrin-3 to the plasma membrane (marked by CD8 or PKC-$\theta$) in 95% CTLs with docked centrosomes (n = 55), indicating that CP110 was not lost from centrioles on association with the plasma membrane (Figure S2). The CP110 binding partner Cep97 also remained associated with mother centrioles docked at the plasma membrane, marked by CD44 (Figure 4D). Docked centrioles retaining Cep97 lacked the daughter centriole marker, centrobin [23], identifying them as mother centrioles (Figure 4E). These results suggest that the CP110/Cep97 complex is not lost from the CTL mother centriole upon membrane docking at the synapse, thus contrasting with the dissociation of these proteins from basal bodies during ciliogenesis.

Taken together, our data show CTLs can polarize the centrosome without exiting the cell cycle. We find CTL mother

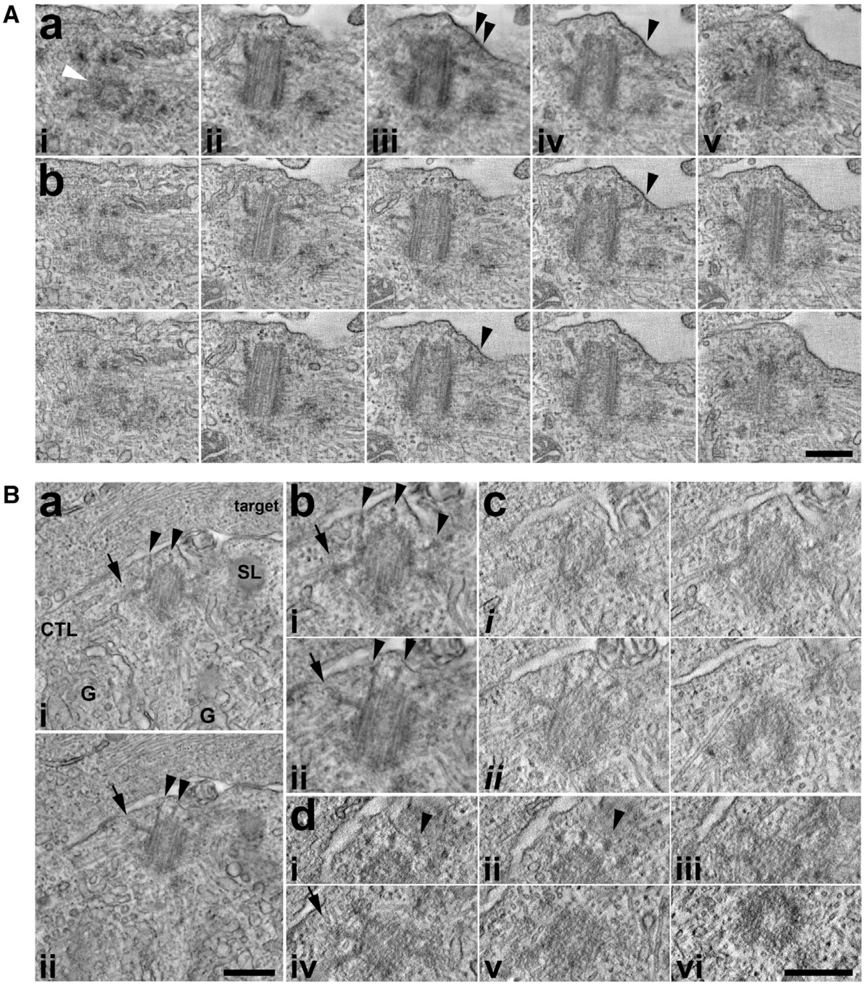


**Figure 3. Docked Mother Centrioles Do Not Form a Transition Zone or Axoneme**

Images from (A) a dual-axis tilt series tomogram of a C57BL/6 CTL-P815 synapse and (B) a single-axis tilt series tomogram of an F5 CTL-EL4 target synapse, shown as multiple (Aa, Ba, and Bb) or paired (Ab, Bc, and Bd) plane projection images aligned (A and Ba–Bc) or tilted (Bd) to the axis of reconstruction. Arrowheads, distal appendages; arrows, sub-distal appendages; G, Golgi complex; SL, secretory lysosome. Scale bars, 300 nm. See also Movies S4 (A), S5 (Ba), and S6 (Bb and Bc).

centrioles dock at the synapse membrane via distal appendages in a manner analogous to mother centriole docking during ciliogenesis but fail to progress to axoneme and transition zone formation. The retention of CP110/Cep97 upon docking is consistent with a block in further ciliogenesis. Interestingly, a recent report showed cilia formation can be induced by CP110 depletion and serum starvation in immune-derived cell lines, suggesting that immune cells have the capacity for ciliogenesis [24]. CP110 levels vary with cell-cycle state with low levels present in quiescent cells and high levels in proliferating cells. It is thought this could prevent aberrant cilia formation during cell division [12, 25]. This raises the possibility that a similar regulation of CP110 in proliferating CTLs might occur, favoring a transient contact of the centrosome with the immunological synapse, allowing CTLs to rapidly repolarize the centrosome and kill multiple targets sequentially.

It has been suggested that polarized release of secretory lysosomes from CTLs may not require the centrosome to contact the membrane [9] but might use short plus-end-directed microtubule motors to reach the synapse [10]. Here, we provide a 3D tomographic analysis showing that the centrosome docks directly at the immunological synapse via the distal appendages of the mother centriole. This organizes the microtubule network directly from the plasma membrane allowing secretory lysosomes to be delivered to the immunological synapse via a minus-end-directed motor alone [1, 4].

Our results not only reveal striking similarities between centrosome docking at the immunological synapse and during early ciliogenesis, but also provide both possible mechanistic and functional explanations as to why cilium formation does not proceed past centrosome docking at the synapse in cytolytic cells.

## EXPERIMENTAL PROCEDURES

### Cell Preparation and Culture

Breeding of genetically modified mice used in this study were licensed by the appropriate UK regulatory authority (Animals, Scientific Procedures Act, 1986), under project license PPL 80/2415, and were approved by the local ethics committee. CTLs from C57BL/6, OT1, and F5 mice, and P815 and EL4 target cells were cultured as in the Supplemental Experimental Procedures or described previously [26, 27].

### TEM and Tomography

CTLs were prepared for TEM as described previously [26, 27]. Thin (50–70 nm) and semi-thin (100–150 nm) sections were used for conventional TEM and thick (400–500 nm) sections used for tomography (see the Supplemental Experimental Procedures).

### Immunofluorescence Microscopy

CTLs and targets were conjugated and adhered to slides at 37°C for 20–30 min, processed, and imaged as in the Supplemental Experimental Procedures. Primary antibodies used were rabbit anti-Cep164 (Novus Biologicals), CP110 or Cep97 (Proteintech); rat anti-CD44 (BD Transduction Labs) or CD8 (YTS192; H Waldmann, Oxford University); and mouse anti-centrobin (Abcam), acetylated tubulin or gamma-tubulin (Sigma-Aldrich), centrin-3 (Novus Biologicals), Lck (Merck Millipore), or PKC-θ (BD Transduction Labs).

### RNAi, Degranulation, and Western Blotting

Non-targeting siRNA and murine Cep83 siRNA were obtained from Dharmacon (ON-TARGET plus Smart pool L-063954 and L-045514). 5 × $10^6$ CTLs were nucleofected with 3 μg of siRNA using Mouse T Nucleofector Kit (Amaxa) and X-001 program according to manufacturer's instructions. Knockdown efficiency and degranulation were assessed 24 hr post-nucleofection as described in the Supplemental Experimental Procedures.

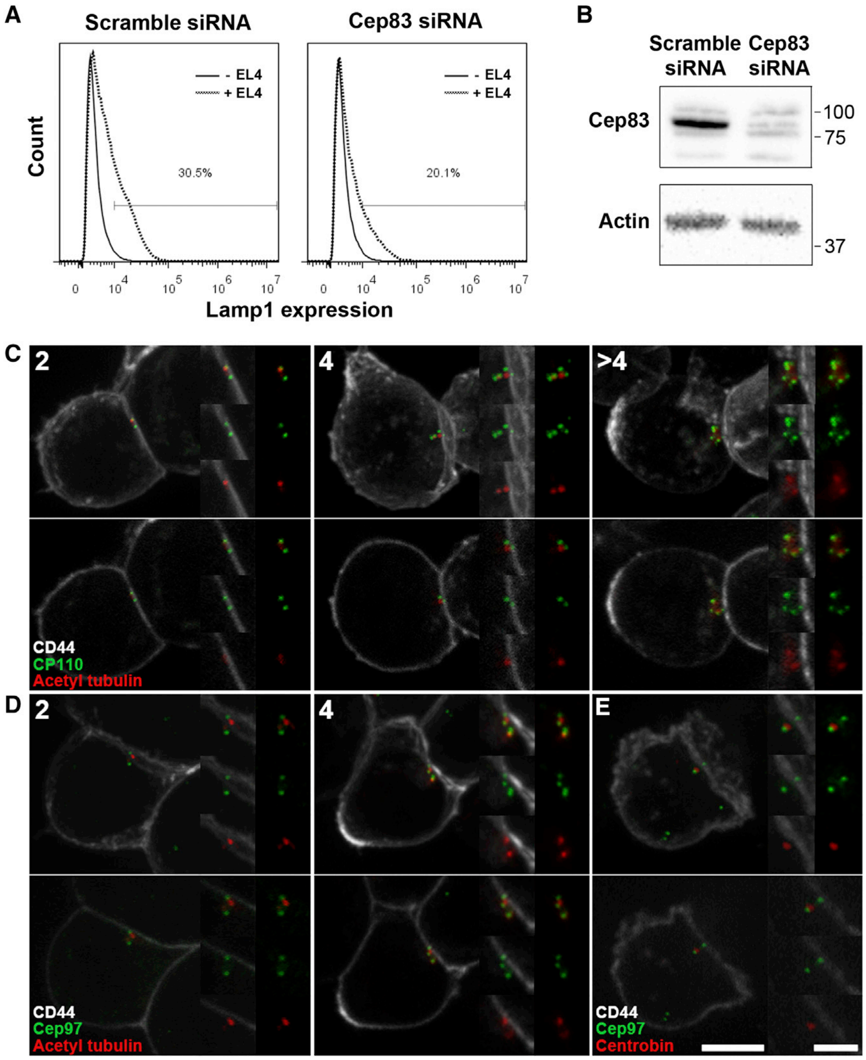


**Figure 4. CTLs Require the Distal Appendage Protein Cep83 for Killing and Retain Cep97 and CP110 on Docking**

(A) Degranulation assay showing Lamp1 appearance on the plasma membrane in OT-I CTLs treated with control (scramble) or Cep83 siRNA, before (–EL4) or after (+EL4) encounter with target cells.

(B) Western blots showing Cep83 and actin upon Cep83 siRNA treatment.

(C–E) Confocal projection (top) and single-section (bottom) images of synapses with centrosomes at the plasma membrane of OT-I (C and D) or C57 CTLs (E) showing CP110 (green; C) and Cep97 (green; D and E) are retained on docked centrioles (acetylated tubulin, red; C and D) in CTLs with two, four, or more than four CP110/Cep97-positive structures, including the docked mother centrioles that lack the daughter centriole marker centrobin (red; E). Scale bars, 5 μm and 2.5 μm (insets). See also Figure S2.

## SUPPLEMENTAL INFORMATION

Supplemental Information includes Supplemental Experimental Procedures, two figures, and six movies and can be found with this article online at http://dx.doi.org/10.1016/j.cub.2015.10.028.

## AUTHOR CONTRIBUTIONS

J.C.S. and G.M.G. designed the study and wrote the paper. J.C.S. carried out all imaging and analysis, with help from J.M.M. and P.V. for tomography acquisition. L.O.R. and K.L.A. carried out siRNA experiments.

## ACKNOWLEDGMENTS

We thank members of the Vaughan lab (Brookes University, Oxford) for assistance with tomography, Alison Schuldt for critical reading of the manuscript, the Wellcome Trust for funding to G.M.G. (075880 and 103930), and CIMR (100140).

Received: November 14, 2014
Revised: August 6, 2015
Accepted: October 13, 2015
Published: December 3, 2015

## REFERENCES

1. Stinchcombe, J.C., Majorovits, E., Bossi, G., Fuller, S., and Griffiths, G.M. (2006). Centrosome polarization delivers secretory granules to the immunological synapse. Nature *443*, 462–465.
2. Stinchcombe, J.C., Salio, M., Cerundolo, V., Pende, D., Arico, M., and Griffiths, G.M. (2011). Centriole polarisation to the immunological synapse directs secretion from cytolytic cells of both the innate and adaptive immune systems. BMC Biol. *9*, 45.
3. Ritter, A.T., Asano, Y., Stinchcombe, J.C., Dieckmann, N.M., Chen, B.C., Gawden-Bone, C., van Engelenburg, S., Legant, W., Gao, L., Davidson, M.W., et al. (2015). Actin depletion initiates events leading to granule secretion at the immunological synapse. Immunity *42*, 864–876.
4. Stinchcombe, J.C., and Griffiths, G.M. (2007). Secretory mechanisms in cell-mediated cytotoxicity. Annu. Rev. Cell Dev. Biol. *23*, 495–517.
5. Griffiths, G.M., Tsun, A., and Stinchcombe, J.C. (2010). The immunological synapse: a focal point for endocytosis and exocytosis. J. Cell Biol. *189*, 399–406.
6. Finetti, F., Paccani, S.R., Riparbelli, M.G., Giacomello, E., Perinetti, G., Pazour, G.J., Rosenbaum, J.L., and Baldari, C.T. (2009). Intraflagellar transport is required for polarized recycling of the TCR/CD3 complex to the immune synapse. Nat. Cell Biol. *11*, 1332–1339.

7. Finetti, F., Patrussi, L., Masi, G., Onnis, A., Galgano, D., Lucherini, O.M., Pazour, G.J., and Baldari, C.T. (2014). Specific recycling receptors are targeted to the immune synapse by the intraflagellar transport system. J. Cell Sci. *127*, 1924–1937.
8. de la Roche, M., Ritter, A.T., Angus, K.L., Dinsmore, C., Earnshaw, C.H., Reiter, J.F., and Griffiths, G.M. (2013). Hedgehog signaling controls T cell killing at the immunological synapse. Science *342*, 1247–1250.
9. Bertrand, F., Müller, S., Roh, K.H., Laurent, C., Dupré, L., and Valitutti, S. (2013). An initial and rapid step of lytic granule secretion precedes microtubule organizing center polarization at the cytotoxic T lymphocyte/target cell synapse. Proc. Natl. Acad. Sci. USA *110*, 6073–6078.
10. Kurowska, M., Goudin, N., Nehme, N.T., Court, M., Garin, J., Fischer, A., de Saint Basile, G., and Ménasché, G. (2012). Terminal transport of lytic granules to the immune synapse is mediated by the kinesin-1/Slp3/Rab27a complex. Blood *119*, 3879–3889.
11. Tanos, B.E., Yang, H.J., Soni, R., Wang, W.J., Macaluso, F.P., Asara, J.M., and Tsou, M.F. (2013). Centriole distal appendages promote membrane docking, leading to cilia initiation. Genes Dev. *27*, 163–168.
12. Spektor, A., Tsang, W.Y., Khoo, D., and Dynlacht, B.D. (2007). Cep97 and CP110 suppress a cilia assembly program. Cell *130*, 678–690.
13. Wheatley, D.N., Wang, A.M., and Strugnell, G.E. (1996). Expression of primary cilia in mammalian cells. Cell Biol. Int. *20*, 73–81.
14. Seeley, E.S., and Nachury, M.V. (2010). The perennial organelle: assembly and disassembly of the primary cilium. J. Cell Sci. *123*, 511–518.
15. Nigg, E.A., and Stearns, T. (2011). The centrosome cycle: centriole biogenesis, duplication and inherent asymmetries. Nat. Cell Biol. *13*, 1154–1160.
16. Tucker, R.W., Pardee, A.B., and Fujiwara, K. (1979). Centriole ciliation is related to quiescence and DNA synthesis in 3T3 cells. Cell *17*, 527–535.
17. Chen, Z., Indjeian, V.B., McManus, M., Wang, L., and Dynlacht, B.D. (2002). CP110, a cell cycle-dependent CDK substrate, regulates centrosome duplication in human cells. Dev. Cell *3*, 339–350.
18. Graser, S., Stierhof, Y.D., Lavoie, S.B., Gassner, O.S., Lamla, S., Le Clech, M., and Nigg, E.A. (2007). Cep164, a novel centriole appendage protein required for primary cilium formation. J. Cell Biol. *179*, 321–330.
19. Sorokin, S. (1962). Centrioles and the formation of rudimentary cilia by fibroblasts and smooth muscle cells. J. Cell Biol. *15*, 363–377.
20. Azimzadeh, J., and Bornens, M. (2007). Structure and duplication of the centrosome. J. Cell Sci. *120*, 2139–2142.
21. Čajánek, L., and Nigg, E.A. (2014). Cep164 triggers ciliogenesis by recruiting Tau tubulin kinase 2 to the mother centriole. Proc. Natl. Acad. Sci. USA *111*, E2841–E2850.
22. zur Stadt, U., Rohr, J., Seifert, W., Koch, F., Grieve, S., Pagel, J., Strauss, J., Kasper, B., Nürnberg, G., Becker, C., et al. (2009). Familial hemophagocytic lymphohistiocytosis type 5 (FHL-5) is caused by mutations in Munc18-2 and impaired binding to syntaxin 11. Am. J. Hum. Genet. *85*, 482–492.
23. Zou, C., Li, J., Bai, Y., Gunning, W.T., Wazer, D.E., Band, V., and Gao, Q. (2005). Centrobin: a novel daughter centriole-associated protein that is required for centriole duplication. J. Cell Biol. *171*, 437–445.
24. Prosser, S.L., and Morrison, C.G. (2015). Centrin2 regulates CP110 removal in primary cilium formation. J. Cell Biol. *208*, 693–701.
25. Tsang, W.Y., and Dynlacht, B.D. (2013). CP110 and its network of partners coordinately regulate cilia assembly. Cilia *2*, 9.
26. Tsun, A., Qureshi, I., Stinchcombe, J.C., Jenkins, M.R., de la Roche, M., Kleczkowska, J., Zamoyska, R., and Griffiths, G.M. (2011). Centrosome docking at the immunological synapse is controlled by Lck signaling. J. Cell Biol. *192*, 663–674.
27. Jenkins, M.R., Stinchcombe, J.C., Au-Yeung, B.B., Asano, Y., Ritter, A.T., Weiss, A., and Griffiths, G.M. (2014). Distinct structural and catalytic roles for Zap70 in formation of the immunological synapse in CTL. eLife *3*, e01310.

Current Biology
Supplemental Information

# Mother Centriole Distal Appendages Mediate Centrosome Docking at the Immunological Synapse and Reveal Mechanistic Parallels with Ciliogenesis

Jane C. Stinchcombe, Lyra O. Randzavola, Karen L. Angus, Judith M. Mantell, Paul Verkade, and Gillian M. Griffiths

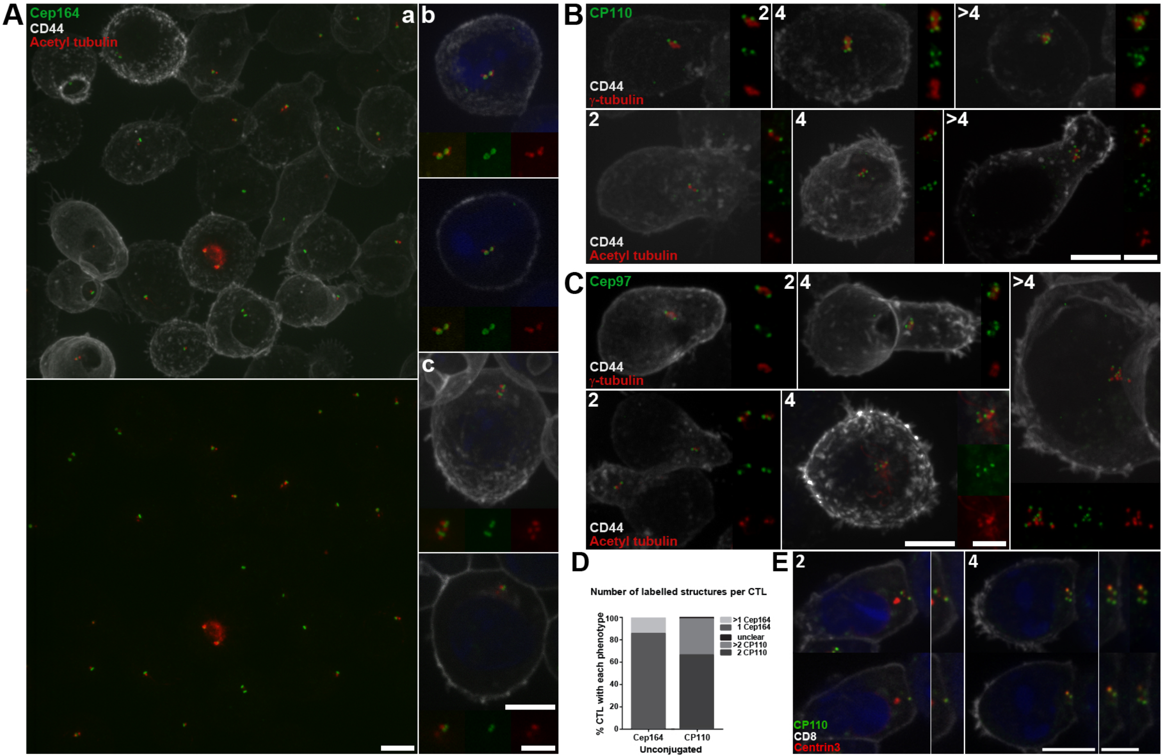
A
Cep164
CD44
Acetyl tubulin
a
b
c
B
CP110
CD44
γ-tubulin
Acetyl tubulin
2
4
>4
C
Cep97
D
Number of labelled structures per CTL
% CTL with each phenotype
>1 Cep164
1 Cep164
unclear
>2 CP110
2 CP110
Cep164
CP110
Unconjugated
E
CP110
CD8
Centrin3

# Figure S1

**Figure S1, related to Figure 1. CTL can contain centrosomes with more than one Cep164-positive mother centriole and/or multiple CP110/Cep97 complexes**

**A. CTL can contain centrosomes with more than one Cep164-positive mother centriole.** Overview (a) and single cell (b-c) confocal images of unconjugated OT-I (a, c) or C57BL/6 (b) CTL labeled with acetylated tubulin (red) for centrioles, the mother distal appendage marker Cep164 (green), and CD44 (white) to delineate the membrane. Multiple-plane projections show total cellular centrosome components (a; top panels b-c) and single plane images show details of the Cep164 labelling (bottom panels b-c). (a) CTL populations contain individual cells with more than one Cep164-positive structure as well as those containing just one. (b-c) Higher power images of the centrioles (insets) show Cep164 is associated with rings characteristic of distal appendage labelling regardless of the number of structures, confirming the presence of multiple mother centrioles in these cells. Nuclei in (b) are stained with Hoechst, blue.

**B-C. CTL can contain 2, 4 or >4 CP110/Cep97 complexes.** Confocal multiple-plane projections images through the region of OT-I (B top row and first panel bottom row; C) or C57BL/6 (B second and third panels bottom row) CTL containing the centrosome, labeled with anti-CP110 (green, B) or Cep97 (green, C), plus CD44 (white) for the surface membrane and either gamma-tubulin for whole centrosomes (red, top rows B and C) or acetylated tubulin for individual centrioles (red, bottom rows B and C). Insets show higher power magnifications of the centriolar region. CP110 (B) and Cep97 (C) have similar distributions in CTL. Cells with 2, 4 or >4 centrosome-associated CP110 (B) or Cep97 (C) positive structures, as indicated, are shown.

**D-E. Analysis of the number of Cep164 and/or CP110-positive structures in CTL. D.** Quantitation of the number of Cep164 or CP110-positive structures/cell using the centriole barrel protein centrin-3 to identify centrioles and CTL-specific synapse membrane markers (CD8, Lck or PKC-theta) to label the CTL cell membrane. **E.** Illustrative images of CTL-target conjugate samples used for quantitation analyses, labeled with CP110 (green), centrin-3 (red) and CD8 (white), and illustrating polarization of 2 and 4 centrioles to the synapse.

Bars: 5 μm, 2.5 μm (insets)

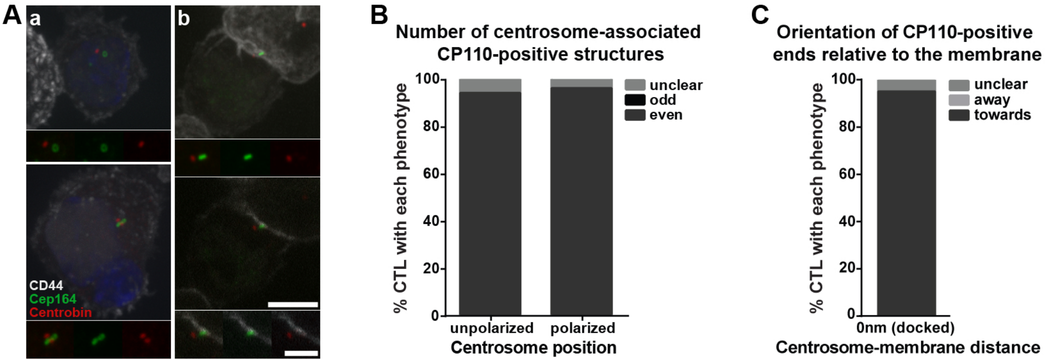
A
a
b
CD44
Cep164
Centrobin
B
Number of centrosome-associated CP110-positive structures
% CTL with each phenotype
100
80
60
40
20
0
unclear
odd
even
unpolarized
polarized
Centrosome position
C
Orientation of CP110-positive ends relative to the membrane
% CTL with each phenotype
100
80
60
40
20
0
unclear
away
towards
0nm (docked)
Centrosome-membrane distance

# Figure S2

**Figure S2, related to Figures 1 and 4. The CP110/Cep97 complex remains associated with the mother centriole on docking**

**A.** Confocal images of OT-1 CTL alone (a, multiple plane projections) or conjugated to EL4 targets (b, multiple plane projection (top) and single plane through the synapse (bottom)) labeled with antibodies against the daughter centriole marker centrobin (red), the mother centriole marker Cep164 (green) and CD44 for CTL and EL4 membranes (white). Higher power images of the centrioles are shown in insets. (a) Centrobin and Cep164 recognise distinct centrioles in cells with one (a, top) and two (a, bottom) centrosomes. (b) The centrobin-positive daughter centriole lies behind the Cep164-positive mother centriole when Cep164 is associated with the contact site membrane (b). Bars: 5 $\mu$m; 2.5 $\mu$m (insets)

**B-C.** Quantitation of samples of CTL-target conjugates prepared for immunofluorescence and quantitation as described for Figures S1D-E and confirming there is no loss of CP110 from CTL centrosomes during killing. (B) The number of CP110 positive structures in polarized versus non polarized cells. (C) The orientation of the CP110-positive distal ends of centrioles at the synapse (defined as CP110-positive end ‘towards’ or ‘away’ from membrane markers on docking).

## Supplemental Experimental Procedures

### Preparation and culture of CTL from C57BL/6 mice and P815 target cells

CTL isolated from C57BL/6 mice were prepared from splenocytes, and stimulated in CTL medium (RPMI 1640, 10% FCS, 1% L-glutamine, 1% sodium pyruvate, 50 µM β-mercaptoethanol, 100 U/ml IL-2 (Roche) and 50 U/mL penicillin/streptomycin (Gibco)) with equal numbers of irradiated (3000 Rad) Balb/c splenocytes at 37°C. CTL were purified over Ficoll Histopaque-1083-1 (Sigma-Aldrich) after 5-6 days and washed before further culture in CTL medium. P815 mouse target cells were maintained in RPMI, 10% FCS and L-glutamine.

### Conjugation of CTL for TEM and TEM tomography

For TEM analysis, CTL were incubated overnight (15–16 hr) in the presence of 1 mg/ml horseradish peroxidase (Serva), washed and resuspended in serum-free RPMI 1640 at 1-2 x $10^6$ cells/ml. Labelled CTL were either used unconjugated or mixed 1:1 with P815 target cells and incubated at 37°C for 20-60 min to form conjugates.  Samples were fixed in 2% paraformaldehyde with either 1.5% or 3% gluteraldehyde, then processed for diaminobenzidine cytochemistry, osmium fixation, urynal acetate staining en bloc and embedding in EPON as previously described [S1-4]. Thin and semi-thin sections were viewed using a Phillips C100 TEM (FEI) at magnifications of 6-11 K. Conventional 2D images were captured on Kodak photographic negative film (Kodak) and the negatives used to generate digital electron micrographs using a Flextight X5 scanner (Hasselblad).

### TEM tomography

Thick (400-500 nm) sections of CTL conjugated to targets as above were collected on film-coated slot grids, either left unstained or stained with lead citrate, and the top and bottom of each section labeled with 15 nm gold (British Biocell International) for 5 min.  Labeled samples were pre-screened using a Phillips C100 TEM to identify cells of interest. Single or dual axis tilt series were collected using an FEI Technai 20, 200 kV TEM equipped with a dual-axis holder (FEI, Eindhoven) with images taken every 1-1.5 degrees from between -70 and -60 degrees to between +60 and +70 degrees at magnifications of between to 15-29 K using the FEI tomography acquisition software (Xplore3D). Tilt series were processed to generate tomographic

reconstructions using the IMOD and 3dMOD packages (Boulder Laboratory for 3-D EM of cells, University of Colorado, USA). Image J software was used for additional image processing and movie generation.

Images shown in Figure 1C represent low (a-b) and higher power (c) projections of multiple (120, a) or pairs of (b-c) sequential planes taken from the reconstructed tomogram in the regions containing the two mother centrioles (a-b, c-bottom) and a procentriole associated with the trailing mother centriole (c-top). Figure 2A shows projections of multiple (190 (a), 50 (b) or 35 (c)) or pairs of (d) sequential planes through different regions of the polarized centrosome. Images in 2B show projections (55 planes, a) or single, non-sequential planes (b-d) from the tomogram series. For Figure 3 each reconstruction is shown as a series of 'thick' (400-500 nm) (Aa, Ba-b) and 'thin' (50-70 nm) (Ab, Bc-d) section images passing through the whole centriole, generated from projections of multiple consecutive planes (50-60 for Aa i-ii and Aa iv-v; 45, 60, 40 or 70 for Ba i, Ba ii, Bb i, Bb ii, respectively) or pairs of consecutive planes (Ab, Bc-d), and organised sequentially. In (A) the series passes left to right from the cartwheel-bearing proximal end of a procentriole (A i, white arrowhead) transversely through the centriole barrel to the other side (A v). For (B), the series passes from the distal side (Bc i-left, Bd i) to the proximal end (Bc ii-right, d vi). (Aa iii) shows a multiple plane projection image through the whole centriole (200 planes). (A) and (Ba-c) are taken from reconstructions shown in made in the xy plane through the z axis. For (B) where the centriole barrel is tilted to the section plane, reconstructions were also made with the x and y axes tilted to align the z-axis with the axis of the centriole barrel (Bd).

**Immunofluorescence and quantitation**

OT-I or C57BL/6 CTL alone or conjugated to EL4 or P815 targets respectively, were either fixed with 4% PFA in PBS, quenched with 15 mM glycine in PBS (20 min) and permeabilized with 0.05-0.2% Triton-X100 (5 min) at RT or fixed and permeabilized with -20°C-cooled methanol for 5 min. Samples were blocked in 1-2% BSA in PBS (>15 min) before labelling with primary antibodies (1 hr at RT or overnight at 4°C), followed by goat or donkey anti-mouse, rat or rabbit Alexa-labeled secondary antibodies (Invitrogen or Jackson Stratech) (1 hr at RT). Samples were washed extensively between each processing step. Nuclei were stained with Hoechst

(1:25,000) in PBS for 5 min and slides mounted with Mowiol or Vectashield (Vector Laboratories Inc.). Samples were analysed using an Andor spinning-disk confocal system (Revolution; Andor) fitted with a spinning-disk unit (CSU-X1; Yokogawa) and Olympus IX81 microscope, and viewed using Olympus 100x and 60x objectives with numerical apertures of 1.40 and 1.42, respectively, and lasers exciting at 405, 488, 543 and 633 nm. Z-stack image series were collected across the depth of the cell containing the total centrosomal content at 0.1 µm intervals and captured using a 512 × 512, 16 $\mu m^2$-pixel camera (iXon; Andor) using IQ software (Andor). Image series were analyzed using Imaris (Bitplane, Switzerland) software to create single plane images and/or projection images of 3D reconstructions and processed with Adobe Photoshop (CS4-CS6; Adobe) software.)

**Quantitation of immunofluorescence samples**

A total of 317 CP110- and 347 Cep164-labeled unconjugated CTL, and 435 CP110- and 320 Cep164-labeled CTL conjugated to targets were quantitated. Samples were co-labeled with primary antibodies against centrin-3, CD8, Lck or PKC-theta and analysed using 3D reconstructions of Z-stack series created using Imaris software. Centrosomes were defined as centrin-3 barrels plus CP110 or Cep164 labeling. Polarization of the centrosome was determined by measuring the shortest distance between the centriole nearest the membrane and the synapse plasma membrane. Orientation of centrioles at the synapse was defined by the position of the CP110-labeled distal end of centrin-3 positive centriole barrels relative to the plasma membrane markers (i.e. end facing ‘towards’ or ‘away’ from the membrane). Orientation of the mother centriole was given by the position (towards or away) of the Cep164-labeled distal end of the Cep164-positive centrin-3-labeled centriole relative to the membrane.

**Degranulation assay and western blotting of siRNA nucleofected cells**

For degranulation assay, CTLs nucleofected with either scramble siRNA or Cep83 siRNA were mixed at 1:1 ratio with EL-4 target cells in 96-well plate at 37°C in the presence of CD107a-PE (LAMP1). After 3 hr of incubation, cells were harvested into cold PBS and resuspended in PBS/0.2% FBS (FACS buffer). Anti CD8-APC was used to differentiate effector CTL and percent of degranulation was measured by analysing Lamp1 expression [S4].

For western blotting CTL were lysed at 2 x $10^7$ cells/ml in lysis buffer (150 mM NaCl, 50 mM Tris-HCl pH 8, 1 mM MgCl2, 2% Triton X-100) supplemented with Protease Inhibitor Cocktail (Roche). Samples were loaded in 2x Laemmli Buffer on 4-12% NuPage Bis-Tris Gel (Invitrogen) under reducing conditions in MES Buffer (Formedium), transferred to nitrocellulose membrane (Amersham) using NuPage transfer buffer (Invitrogen) and incubated in TBS, 5% milk, 0.05% Tween-20 (Sigma) with rabbit anti-Cep83 and mouse anti-actin (Sigma) primary antibodies followed by anti-rabbit IgG-horseradish peroxidase and anti-mouse IgG-horseradish peroxidase (Thermo Fisher Scientific) secondary antibodies. Blots were developed with ECL Prime reagent (Amersham) and signal was recorded on a ChemiDoc MP Imager (Biorad).

## Supplemental References


S1. Stinchcombe, J.C., Salio, M., Cerundolo, V., Pende, D., Arico, M., and Griffiths, G.M. (2011). Centriole polarisation to the immunological synapse directs secretion from cytolytic cells of both the innate and adaptive immune systems. BMC Biology *9*, 45.

S2. Stinchcombe, J.C., Barral, D.C., Mules, E.H., Booth, S., Hume, A.N., Machesky, L.M., Seabra, M.C., and Griffiths, G.M. (2001). Rab27a is required for regulated secretion in cytotoxic T lymphocytes. The Journal of Cell Biology *152*, 825-834.

S3. Stinchcombe, J.C., Bossi, G., Booth, S., and Griffiths, G.M. (2001). The immunological synapse of CTL contains a secretory domain and membrane bridges. Immunity *15*, 751-761.

S4. Jenkins, M.R., Tsun, A., Stinchcombe, J.C., and Griffiths, G.M. (2009). The strength of T cell receptor signal controls the polarization of cytotoxic machinery to the immunological synapse. Immunity *31*, 621-631.
